# Genomic and Physiological Insights Into Heat–Drought Tolerance in Wheat Through GWAS and Phenotypic Evaluation

**DOI:** 10.1111/pce.70510

**Published:** 2026-04-22

**Authors:** Jingjuan Zhang, Weinan Xu, Rakshith S. R. Gowda, Joel Johnstone, Malona Alinsug, Abhishek Bohra, Vanika Garg, Annapurna Chitikineni, Dion Bennett, Meixue Zhou, Meiqin Lu, Chengdao Li, Zhong‐Hua Chen, Reyazul Rouf Mir, Rajeev K. Varshney

**Affiliations:** ^1^ Centre for Crop and Food Innovation, WA State Agricultural Biotechnology Centre, Food Futures Institute Murdoch University Murdoch Western Australia Australia; ^2^ Philippine Nuclear Research Institute – Department of Science & Technology Quezon City Philippines; ^3^ Australian Grain Technologies Northam Western Australia Australia; ^4^ Tasmanian Institute of Agriculture University of Tasmania Launceston Tasmania Australia; ^5^ Australian Grain Technologies Narrabri New South Wales Australia; ^6^ School of Agriculture, Food and Wine, Waite Research Institute University of Adelaide Glen Osmond South Australia Australia

**Keywords:** drought stress, genome‐wide association study, heat and drought tolerant genotypes, heat stress, pre‐breeding, Triticum aestivum L, yield traits

## Abstract

Climate change‐driven heat and drought stresses during reproductive stages significantly threaten wheat productivity. To investigate the genetic and physiological basis of combined heat–drought (HD) tolerance, we evaluated 345 wheat genotypes under three environments of HD stresses, non‐stress glasshouse conditions and a late‐sowing field trial. HD stresses caused significant reductions in chlorophyll content, flag leaf area, biomass, seed‐setting rate and grain weight‐related traits. Notably, HD‐tolerant lines maintained higher grain weight, grain number and chlorophyll retention, with less than half the reductions observed in sensitive genotypes. A genome‐wide association study using a 40K single‐nucleotide polymorphism (SNP) array identified 124 candidate SNPs (cSNPs) associated with 51 traits across three environments with 78 cSNPs associated with HD tolerance. In total, 24 cSNP blocks exhibited pleiotropic associations with multiple traits under those three environments. Tight genomic co‐localisations were detected between chlorophyll content (SPAD or CCM200 values), flag leaf width, seed‐setting rate and grain yield components (thousand grain weight, grain number per spike), with superior haplotypes identified, supporting their utility in selections. Stay‐green traits appeared to contribute significantly to yield stability under HD stresses. Those results provide valuable genomic and physiological insights into wheat HD tolerance for future targeted wheat breeding.

## Summary statement

Stable chlorophyll content and flag leaf green area are reliable indicators for selecting high‐yielding wheat under HD stresses, as evidenced by their strong QTL linkages in GWAS. The superior haplotypes and HD‐tolerant genotypes identified will accelerate HD stress‐resilient wheat breeding.

## 1 Introduction

Wheat is a staple food source for approximately 35% of the world's population. It is cultivated on about 17% of the world's arable land ( ~ 220 million hectares), producing around 750 million tonnes of grains annually (Erenstein et al. 2021). In Australia, wheat production reaches approximately 22 million tonnes each year, with Western Australia contributing about 40% of the national total (Farias and Scanlon 2023), with a gross value exceeding AU$6 billion. Over 75% of Australian wheat is exported, accounting for 11% of the global wheat trade (Erenstein et al. 2022). However, climate change—particularly the increasing frequency of extreme weather events—has intensified the occurrence of heat waves and droughts, severely impacting wheat growth and productivity worldwide (Zahra et al. 2021). Heat and drought (HD) stresses can reduce wheat grain yield (GY) by up to 69% and 86%, respectively (Prasad et al. 2011; Yang et al. 2021; Kumar et al. 2022). Globally, each 1°C rise in temperature leads to an estimated 6% reduction in wheat yield (Zhao et al. 2017), and in Australia, a 2°C temperature increase could slash GY by as much as 50% during a typical growing season (Asseng et al. 2011).

Heat stress occurs when plants are exposed to temperatures above their optimal range, resulting in irreversible damage to growth and development (Wahid et al. 2007). The optimal temperature range for wheat development is 17°C–23°C, with temperatures between 31°C and 35°C known to cause serious damage, particularly to flowering (due to pollen abortion) and grain filling (Porter and Gawith 1999). Between 30°C and 35°C, the respiration rate in wheat plants increases rapidly, while the photosynthetic rate declines significantly. At higher temperatures, respiration eventually diminishes due to damage to the respiratory apparatus (Zheng et al. 2025). Elevated temperatures increase evapotranspiration, leading to higher vapour pressure deficits and water scarcity (Gobin 2012; Lobell et al. 2013). This water deficit diminishes canopy cooling via evaporation, further exacerbating heat‐induced damage. Heat stress accelerates plant development, reduces tillering and floret fertility and impairs grain set and carbohydrate assimilation during grain filling (Shirdelmoghanloo et al. 2022). HD stresses significantly reduce both the grain number and individual grain weight in wheat, although the timing of stress determines which trait is more affected (Prasad et al. 2011; Qaseem et al. 2019). Water deficit inhibits photosynthesis, while heat stress leads to oxidative damage to photosynthetic machinery and cell membranes (Farias and Scanlon 2023).

When wheat plants experience combined HD stresses after anthesis, grain number per spike may remain stable, but thousand‐grain weight (TGW) is often significantly reduced. This is due to limitations in carbon translocation to developing grains, resulting in smaller grains and lower yields (Zahra et al. 2021; Ullah et al. 2022). Globally, HD stresses typically coincide during anthesis and grain filling, the most vulnerable stages of wheat development, leading to substantial yield losses (Zahra et al. 2021; Kumar et al. 2022). These stresses also increase ovule and floret abortion (Barnabás et al. 2008; Fábián et al. 2019; Zahra et al. 2021), significantly reducing the number of florets and grains per spike. In general, HD stresses lead to chlorophyll degradation, reduced photosynthetic capacity, accelerated senescence and shortened grain‐filling duration (Kumar et al. 2022). This, in turn, decreases sucrose transport to the spike, potentially resulting in ovule and floret abortion (Barnabás et al. 2008). During or following HD conditions, a significant reduction in leaf photosynthates causes plants to rely on stored carbon reserves, such as stem water‐soluble carbohydrates (WSCs), for grain filling. The contribution of stem WSCs to grain development depends on their storage capacity and remobilisation efficiency (Farooq et al. 2018). Meanwhile, some tolerant wheat genotypes can maintain leaf greenness under stress through various mechanisms. For example, certain tolerant genotypes regulate stomatal conductance and transpiration rates via the efflux of K^+^ ions to sustain photosynthesis (Abbas et al. 2025); others preserve membrane fluidity and stability—thereby reducing damage to cellular components—by altering the ratio of saturated to unsaturated fatty acids or by increasing phosphatidic acid and phosphatidylserine levels (Yu et al. 2025). Significant changes in the abundance of proteins (e.g., accumulation of misfolded proteins) in flag leaf were found to be associated with chlorophyll synthesis, carbon fixation, protein turnover and redox regulation under heat stress (Rampino et al. 2009; Lu et al. 2017; Sun et al. 2022). A key focus in breeding heat‐ and drought‐tolerant wheat is the identification of ‘stay‐green’ genotypes capable of sustaining physiological functions during stress. Stay‐green traits are well‐documented as being positively associated with tolerance to HD (Kumar et al. 2022). Tolerant wheat varieties tend to preserve chlorophyll content for extended periods and maintain photosynthetic capacity under stress conditions (Kamal et al. 2019). In several cereal crops, stay‐green traits have been used as selection criteria for genetic improvement in HD tolerance (Christopher et al. 2016; Christopher et al. 2018; Taria et al. 2023). These traits are strongly associated with the maintenance of green leaf area after exposure to HD. A reduction in green leaf area due to stress can lower photosynthetic assimilates, resulting in yield loss. Conversely, the sustained presence of green leaf area is considered an indicator of stress tolerance (Senapati et al. 2018).

Genome‐wide association studies (GWAS), based on linkage disequilibrium, have been successfully employed to dissect the genetic basis of agronomic traits (Srivastava et al. 2020; Alseekh et al. 2021), and have become an effective tool for gene discovery and marker development for complex traits (Zhu et al. 2008). Utilising diverse germplasm, GWAS enables the identification of superior alleles and the elucidation of complex genetic mechanisms involved in biotic and abiotic stress tolerance in wheat (Oyiga et al. 2018; Hu et al. 2021). However, GWAS in wheat has lagged behind that in other crops such as rice and maize, primarily due to wheat's genomic complexity and the high proportion of repetitive sequences (Li et al. 2019). Recent advancements in SNP arrays and accurate resequencing technologies have produced high‐resolution genetic maps, accelerating GWAS research in wheat (Devate et al. 2022; Govta et al. 2022). Quantitative trait loci (QTL) associated with stay‐green traits have been identified on chromosomes 7DS, 3BS and 1AS (Kumar et al. 2010). In another study, three QTLs were detected on chromosomes 2D, 4A and 4D, with the 4D QTL co‐locating with the semi‐dwarfing gene *Rht‐D1*. The stay‐green trait co‐segregates with the wild‐type (tall) Rht‐D1a allele, highlighting the drought tolerance of tall plants under rainfed conditions (Cook et al. 2021). Furthermore, the QTLs for chlorophyll content were detected on 1A, 1B, 2A, 2B, 2D, 3B, 4A, 4B, 4D, 5A, 5B, 5D, 7A and 7B (Talukder et al. 2014; Maulana et al. 2018; Zhai et al. 2021; Chen et al. 2025). In terms of GY, the genetic loci of heat tolerance were detected on chromosomes 1A, 1B, 2A, 2B, 2D, 3A, 3B, 4A, 5A, 5B, 6B, 6D, 7A and 7B in wheat and durum wheat (Mason et al. 2011; Shirdelmoghanloo et al. 2016; Hassouni et al. 2019; Li et al. 2019; Sun et al. 2022). Nevertheless, QTL analyses across multiple and diverse environments remain limited (Li et al. 2019). Therefore, identifying stable QTLs and alleles for agronomic traits under contrasting environmental conditions remains a key objective for wheat breeding. Selection of heat‐tolerant genotypes and associated physiological traits represents an effective strategy to improve wheat productivity under hot and dry environments.

In this study, we aimed to identify wheat genotypes tolerant to combined HD stresses, elucidate the physiological and genetic mechanisms underlying their resilience during the anthesis stage, and identify candidate single‐nucleotide polymorphisms (SNPs) associated with phenotypic traits. Therefore, we hypothesised that genotypes exhibiting a stay‐green phenotype following HD treatment are more likely to be associated with stress tolerance. To test this hypothesis, a diverse panel of 345 wheat genotypes was evaluated under controlled HD stress conditions and a late‐sown field trial. We assessed yield losses and physiological responses, characterised genetic variation in response to stress and performed genome‐wide association analyses to identify trait‐associated loci. Our findings provide key insights into the physiological and genetic basis of stress resilience and offer potential selection criteria for breeding wheat varieties with enhanced tolerance to HD stresses.

## 2 Materials and Methods

### 2.1 Plant Materials

HD stress experiments were conducted at Murdoch University (MU), Perth, Australia, in 2023, using both a heat chamber and a glasshouse, along with a late sowing field trial in Northam, Western Australia (31.383135° S, 116.572613° E), Australia. A total of 345 wheat genotypes—including 319 commercial cultivars and landraces, 19 double haploid lines selected based on superior field performance and their 7 parental lines—were evaluated in the glasshouse experiments. Of these, 330 genotypes were used in the field trial based on seed availability. Among the 319 commercial lines, 195 were sourced from The University of Tasmania, Launceston, Australia and 124 from Australian Grain Technologies (AGT), Australia (Table S1).

### 2.2 Glasshouse Trial

In the glasshouse, a total of 345 genotypes were subjected to two treatments: (i) a combined heat–drought (HD) stress treatment at 36°C (day)/22°C (night) during anthesis for five consecutive days, and (ii) a control condition. Each treatment had four replicates. The design consisted of 345 genotypes × 2 treatments × 4 replicates = 2760 pots. Each pot (L90 mm × W90 mm × H170 mm) was filled with 0.74–0.75 kg of a standardised potting mix containing 65 g NPK and 100 g Osmocote slow‐release fertiliser per 40 L of soil. Genotypes were grouped into 11 classes based on plant height (PH) and randomised within each group by Analytics for the Australian Grains Industry (AAGI) at Curtin University, Perth, Australia. Sowing occurred between 31 May and 3 June 2023 (Figure S1a), with two seeds per pot. At the three‐leaf stage (23 June), seedlings were thinned to retain one plant per pot. Plants were irrigated to field capacity daily via watering mats until anthesis. At anthesis, control plants remained in the glasshouse. HD‐treated plants were removed on the day of anthesis (based on the main spike), placed on trays with water and transferred to the heat chamber the next day. The heat chamber ramped up the temperature starting at 9:00 a.m. (1°C per 5 min) to reach 36°C ± 2°C–3°C by ~10:30 a.m., and cooling began at 4:00 p.m. Plants were kept under HD stresses without watering for 5 days, then weighed, and allowed 1 week of recovery on trays with water before being returned to the watering mats under standard glasshouse conditions (Figure S1b–g).

### 2.3 Field Trial

The field trial in Northam (WA) included 330 of the 345 genotypes evaluated in the glasshouse trial. These genotypes were sown from 19 to 21 June 2023 to coincide with naturally occurring heat stress in Western Australia, Australia. Genotypes were randomised in three replicates, each consisting of 11 ranges (33 total), with 32 rows per range (1 m row length, 0.3 m inter‐row spacing). Machinery access was accommodated by 1.2–1.8 m gaps between ranges (Figure S1h). The trial was managed by AGT using local best practices. An XPS‐2M weather station was installed adjacent to the field to monitor temperature and rainfall. Due to late sowing, the average anthesis date was 2 October 2023, which is different from the normal wheat flowering period of around the end of August to the 20th of September. From 1 September onward, ambient temperatures ranged from 17°C to 35°C, with extremes of 42.34°C (max) and 0.31°C (min) (Figure S1i). Significant heat waves occurred on 28 September (35.78°C), 17 October (38.19°C) and 25 November (42.37°C). Total rainfall during September–November was 60.9 mm, with October (4.18 mm) and November (15.72 mm) being particularly dry.

### 2.4 Temperature and Soil Moisture Monitoring

Temperature loggers recorded air temperatures every 15 min in both the heat chamber and glasshouse from anthesis until harvest (Figure S1d,e). Glasshouse temperatures ranged from 10.0°C to 30.0°C, while the heat chamber reached 15.0°C–49.9°C. Although the heating system was set to a maximum of 36°C, actual temperatures exceeded 45°C, indicating more severe heat stress than anticipated. Due to a battery failure, temperature data from the heat chamber were unavailable prior to 10 October 2023; however, October–December patterns were used to infer earlier trends.

Soil in all pots was standardised by weight before sowing. Each pot's weight was recorded before and after HD treatment. Control pots were weighed at anthesis. The soil of three pots was oven‐dried at 105°C for 3 days post‐harvest to estimate dry soil mass. After subtracting pot weight, soil water content was calculated through the following formula:

$$\text{Soil water content}\;(\%)=\frac{W_{\text{moist soil}}-W_{\text{dry soil}}}{W_{\text{dry soil}}}\times 100$$

Soil water retention properties were derived from prior data (Zhang 2008; Zhang et al. 2009; Zhang et al. 2015), with water content at field capacity and permanent wilting point estimated at 109% (*Ψ*soil = –0.01 MPa) and 33% (*Ψ*soil = –1.5 MPa), respectively.

### 2.5 Phenotypic Measurements

HD‐treated plants were measured at three time points: anthesis, day 5 of HD stresses, and 1‐week post‐stress (12 days after anthesis). Measurements included CCM200 (Apogee Instruments, USA for chlorophyll content), and flag leaf SPAD, leaf temperature, linear electron flow (LEF), and leaf thickness using the PhotosynQ platform (PhotosynQ Inc., East Lansing, MI, USA). Leaf length and width were recorded to calculate flag leaf area (length × width × 0.83) (Chen et al. 2019). Control plants were similarly measured at anthesis, with ~60 plants re‐evaluated 2 weeks later. However, due to negligible differences, control data at anthesis were used for analysis. In the field, the anthesis date and flag leaf chlorophyll content were recorded at anthesis using CCM‐200.

### 2.6 Pre‐ and Post‐Harvest Data Collection

In the glasshouse, PH, stem length, peduncle length, spike length, tiller number and spike number were recorded during harvesting. Main and sub‐tiller spikes were harvested and weighed separately to determine seed‐setting parameters. After approximately 1 month of air drying in the glasshouse, the biomass of each whole plant was measured. Due to labour constraints, spike and seed traits were analysed from three replications per treatment. These traits included grain weight per plant; spikelet number per spike; floret number per spike (the number of florets with fully developed glume and lemma); grain number per spike; and seed‐setting rate per spike (calculated as grain number divided by floret number); TGW; and seed plumpness. Plumpness was assessed as the mass percentage of grain > 2.8 mm in diameter (Singh et al. 2012), while seeds < 2.0 mm were considered undersized (Blakeney et al. 2011). Other seed parameters, including seed length, seed width, seed thickness, seed roundness, average seed area, aspect ratio and mini test weight, were measured using SeedCount SC5000 (Next Instruments, Condell Park, NSW, Australia). The relative data of HD versus control for each phenotype were used to evaluate genotype performance. In addition, in the field, PH was measured before harvest in November 2023. Plants were hand‐harvested and threshed with AGT assistance. The field data of GY, PH, days to anthesis (anthesis), flag leaf chlorophyll content (CCM200) and TGW were used (Table S2).

### 2.7 Genotyping

Of the 345 genotypes, genomic DNA was extracted from the young leaves of a single plant per genotype for 319 genotypes using the cetyltrimethylammonium bromide method. The DNA quantity and quality met the requirements for genotyping using the Infinium Wheat Barley 40K v1.1 BeadChip assay (Illumina Inc., USA). A total of 26,139 SNPs were initially scored. After filtering SNPs with minor allele frequency < 5%, heterozygosity > 10%, missing data > 10%, or unknown chromosomal position, 15,429 high‐quality SNPs were retained for GWAS. SNP positions were based on the Chinese Spring reference genome v2.1 (IWGSC RefSeq v2.1).

### 2.8 Genome‐Wide Association Analysis

Population structure of the 319 accessions was assessed using STRUCTURE 2.3.4 with 15,429 SNPs. Simulations were run for *K* = 1–10 with five iterations each, using 100,000 burn‐in and 100,000 MCMC repetitions. Principal component, cluster heatmap and chromosome SNP density were analysed using software R. A neighbour‐joining tree was constructed using MEGA 11 based on genome‐wide SNP data at 1 Mb intervals.

While GWAS is widely recognised as a powerful approach for dissecting complex traits in crop species (Tibbs Cortes et al. 2021), a major challenge associated with GWAS is the high incidence of false‐positive associations, which can obscure meaningful biological signals. To address this, we employed three complementary statistical models—Fixed and random model Circulating Probability Unification (FarmCPU), General Linear Model (GLM) and Mixed Linear Model (MLM)—to minimise false discovery rates.

The rMVP package in R was used in GWAS (Yin et al. 2021), applying three models of GLM, MLM and FarmCPU. FarmCPU was used as the primary model due to its robustness (Kaler et al. 2020). Significance thresholds were set at *p* < 1.88 × 10⁻⁵ (–log₁₀*P* > 4.7) for FarmCPU and GLM, and *p* < 0.0001 (–log₁₀*P* > 4) for MLM in accordance with established practices (Li et al. 2019). PCA was also performed in R. Manhattan, and quantile–quantile (Q‐Q) plots were generated to visualise marker–trait associations (MTAs) and evaluate model fit. MTAs that were consistently identified in at least two of the three models were considered robust and statistically significant. This cross‐validation strategy enhanced the reliability of detected associations and mitigated false positives.

Several key associations validated the biological relevance of this approach. For instance, a candidate SNP (cSNP) block on chromosome 4D (designated 4D‐1) was associated with PH across all three environments and included the well‐known semi‐dwarfing gene *Rht2*, suggesting it as a likely causal gene for the observed phenotype. Similarly, the 5A‐3 cSNP block harboured *Vrn1‐5A*, which was significantly associated with TGW under HD stresses, indicating a potential regulatory role in grain filling. On chromosome 5B, a cSNP linked to flowering time (anthesis_F) was located 5.1 Mb upstream of *Vrn1‐5B*, while the 5D‐1 cSNP associated with flowering in glasshouse conditions (anthesis_GH) was only 1.3 Mb upstream of *Vrn1‐5D*. These findings further support the biological plausibility and robustness of the identified associations.

### 2.9 Candidate Gene Prediction and Haplotype Analysis

To identify putative genes underlying the significant MTAs, a representative candidate SNP (cSNP) was selected for each association based on the highest LOD score across the three GWAS models. Closely positioned cSNPs (within a 3 Mb window) were grouped into cSNP blocks. Genomic regions corresponding to these cSNP blocks were examined to identify potential candidate genes linked to the observed phenotypes. Gene mining was conducted using the IWGSC RefSeq v2.1 wheat genome assembly and gene annotation available through the International Wheat Genome Sequencing Consortium (http://www.wheatgenome.org/).

The haplotype analysis was conducted using 319 genotypes on the cSNP blocks and surrounding SNPs associated with HD‐tolerant traits, particularly chlorophyll content‐related traits (CCM200, SPAD, LEF); leaf area, including leaf width and length; key GY‐related traits, such as days to anthesis, GWperPlant or per main/sub‐tiller spike, grain number per spike, TGW and seed‐setting rate under HD. The haplotype groups were defined based on the combined SNP alleles, and superior haplotypes were identified according to the phenotypic performance of each group. Tukey's post hoc (SPSS) was performed to assess statistical significance among groups. Haplotype groups with fewer than three accessions were excluded from the comparison.

### 2.10 Statistical Analysis

Phenotypic data were analysed using univariate analysis of variance within the GLM framework implemented in IBM SPSS v30 (California State University Information Services, Los Angeles, USA). Significant differences among genotype groups were further examined using one‐way ANOVA followed by Tukey's Honest Significant Difference (HSD) post‐hoc tests. Pearson correlation coefficients among traits were calculated using the rcorr function in the R package Hmisc (Patterson and Thompson 1971). Data visualisation—including boxplots, violin plots, principal component analysis (PCA) and PCA biplots—was performed using the ggplot2 package in R. Broad‐sense heritability (*H* ^2^) for each trait under different treatments was estimated using linear mixed‐effect models implemented in the lme4 package in RStudio.

## 3 Results

### 3.1 Effects of HD Treatment on Physiological Traits

Under controlled conditions in the glasshouse trial, 345 wheat genotypes were subjected to combined HD treatment. The HD treatment substantially reduced soil water content from 72.1% to 22.1%, well below the wilting point threshold of 33% (Figure S1f), thereby confirming the effective imposition of simultaneous HD stresses. HD treatment had a significant impact on most genotypes (Tables S3–S4). However, a few genotypes exhibited complete recovery with minimal phenotypic damage following HD stresses (Figure S1g). Substantial genotypic variations were observed for all traits, and significant treatment effects were detected for most measured traits, except for leaf length, spikelet number per sub‐tiller and seed‐setting rate of the main spike. Several traits did not show notable genotype‐by‐environment interactions, including tiller number and spike number per plant, PH, stem length, spike length, leaf length, leaf area, seed roundness and thickness, and < 2.0 mm grain size class (Table S4). Post‐recovery chlorophyll measurements (SPAD values) of flag leaves showed that 70 genotypes maintained SPAD > 20 in at least two of four replicates, with 50 genotypes recovering to > 80% and 20 to 50%–80% of their respective control levels (Table S5).

Morphological measurements revealed significant post‐HD declines in PH (–2.8%) and stem length (–3.3%) (*p*  <  0.05), while peduncle length decreased by 5% (*p*  <  0.001). Interestingly, spike length slightly increased by 1.8% (*p*  <  0.05) (Figure S2).

**Figure 1:**
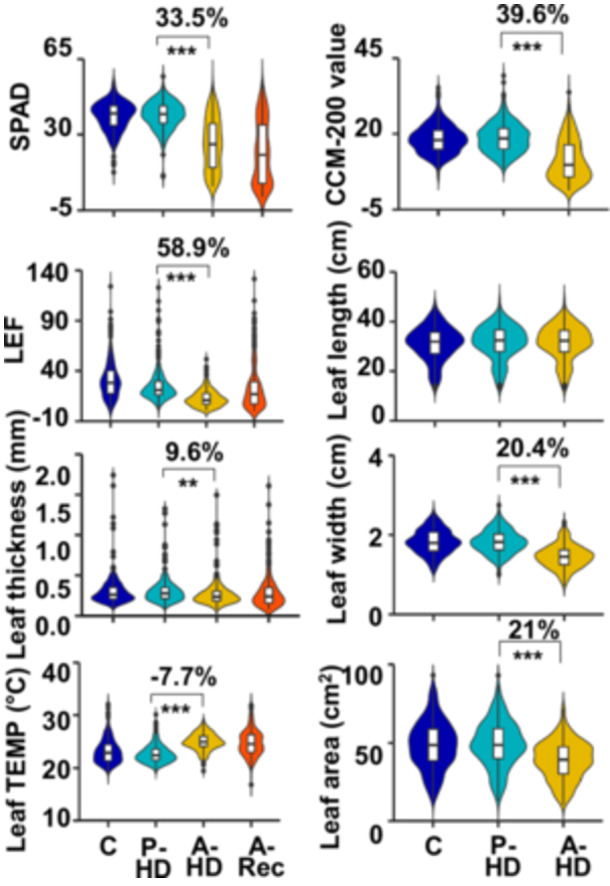
The deduction of SPAD (chlorophyll content), linear electron flow (LEF, *µ*mol electrons m^−2^ s^−1^), flag leaf thickness, leaf area and leaf width and an increase of flag leaf temperature (TEMP). Treatments: control (C) filled with dark blue, followed by before HD (P‐HD) with light blue, after 5 days HD (A‐HD) with yellow and after 7 days recovery (A‐Rec) with red. **p < 0.01; ***p < 0.001.

Furthermore, HD stresses often markedly accelerates leaf senescence (Tan et al. 2023). In this study, leaf‐level physiological traits were assessed using PhotosynQ and CCM200, and showed normally distributed values (Figure 1). Compared to control and pre‐stress conditions, significant post‐HD reductions were observed in SPAD (–33.5%), CCM200 (–39.6%) and LEF (–58.9%). Flag leaves became 9.6% thinner, while leaf temperature rose by 7.7%. Leaf area decreased by 21.0% (*p*  <  0.001), primarily due to a 20.4% reduction in leaf width, as leaf length remained unchanged.

### 3.2 Effects of HD Treatment on Yield and Grain Traits

**Figure 2:**
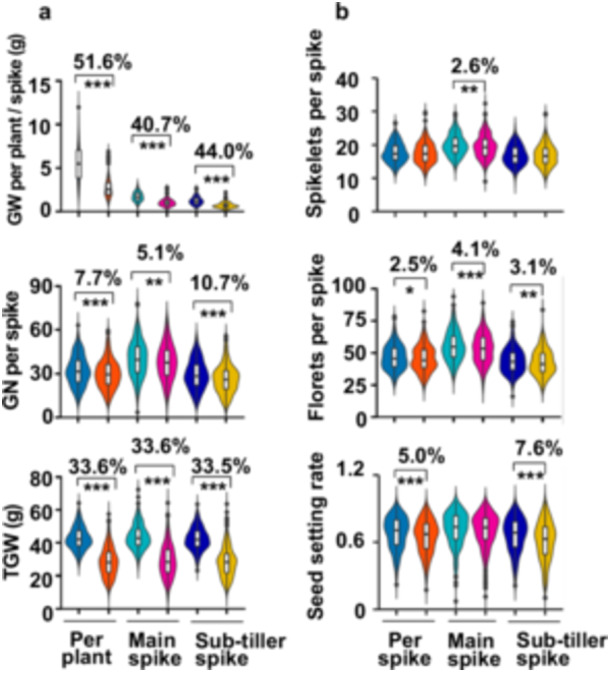
The changes in key yield phenotypes of (a) grain weight (GW), grain number (GN) per spike, thousand grain weight (TGW) for the whole plant, the main spike and sub‐tiller spike, (b) spikelets and florets per spike, and seed‐setting. Treatments: control filled with blue; and HD treated filled with warm colours (red, pink and yellow). *p < 0.05; **p < 0.01; ***p < 0.001. [Color figure can be viewed at wileyonlinelibrary.com]

In the glasshouse, HD stresses caused a pronounced decline in yield‐related parameters. Grain weight per plant declined by 51.6% (Figure 2a), primarily due to a 33.6% reduction in TGW. Grain number per spike decreased by 7.7%. Grains from both main and sub‐tiller spike exhibited substantial weight losses, with sub‐tiller spikes experiencing a slightly greater reduction (3%), likely due to a more pronounced decline in grain number (Figure 2a). Spikelet number per spike declined significantly in main spikes but not in sub‐tillers. Floret number per spike was also reduced in both, with the main spike showing a 1% higher decrease. A substantial reduction (–7.6%) in seed‐setting rate was observed in sub‐tiller spikes, resulting in an average per‐tiller reduction of up to 5% after HD treatment (Figure 2b). Biomass decreased by 26.9%, and total spike weight by 44.4%. Main spike weight decreased by 34.1%, and the average single sub‐tiller spike weight was reduced by 35.2%, which was slightly greater (1.1%) than the reduction in main spike weight. Tiller number and total spike number were also significantly decreased (Figure S3). Mini test weight dropped by 33.8%. Grain size and morphology were also affected. Seed width, thickness, area and roundness decreased by 13.2%, 30.9%, 15.6% and 20.4%, respectively, while seed length was minimally reduced (1.3%). This led to a 15.1% increase in seed aspect ratio. Grain size distribution shifted toward smaller seeds, with a 57.3% reduction in the > 2.8 mm size class and a 99.8% increase in the < 2.0 mm class (Figure S4a,b).

#### 3.2.1 Correlations Among Phenotypic Traits Under HD Treatment

**Figure 3:**
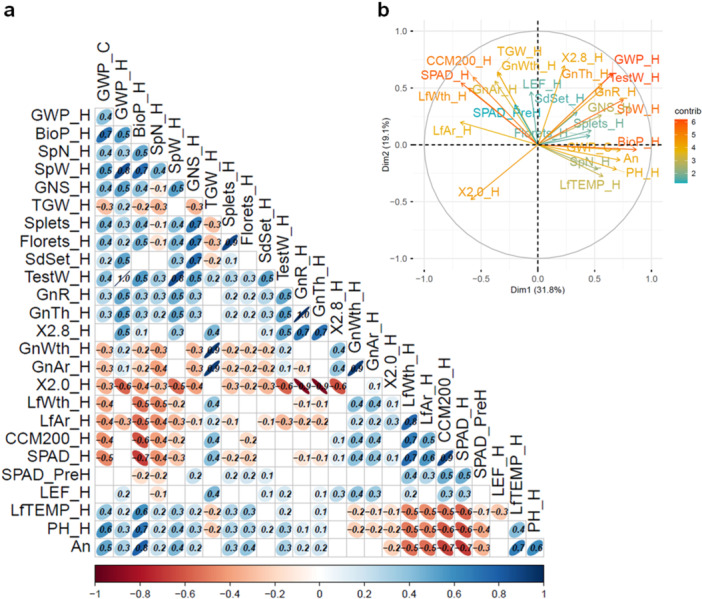
Phenotypic correlations and PCA analysis between yield‐related traits. The correlations (a) and PCA (b) were on the absolute parameters after the HD treatment. The values are correlation coefficients r; The areas and colours of ellipses show the absolute value of corresponding r. Right and left oblique ellipses indicate positive and negative correlations, respectively. The values without ellipses indicate insignificance at the 0.05 level. _C, under control; _H, HD; An, days to anthesis; BioP, dry biomass per plant; CCM200, the value of CCM200; Florets, average florets per spike; GnAr, average seed area; GnR, seed roundness; GNS, average grain number per spike; GnTh, average seed thickness; GnWth, average seed width; GWP, grain weight per plant; LEF, linear electron flow; LfAr, flag leaf area; LfTEMP, flag leaf temperature; LfWth, flag leaf width; PH, plant height; SdSet, seed‐setting rate per plant; SPAD_PreH, SPAD value before HD treatment; SPAD, the value of SPAD; Splets, average spikelet per spike; SpN, spike number per plant; SpW, total spike weight per plant; TestW, mini test weight; TGW, TGW per plant; ×2.0, < 2.0 mm screen; ×2.8, > 2.8 mm screen. [Color figure can be viewed at wileyonlinelibrary.com]

Significant correlations were observed among the traits under HD treatment, as shown in Figure 3a, where grain weight was strongly linked with test weight (*r*  = 1.0), spike weight (*r*  =  0.8) and dry biomass, grain number, seed setting, grain roundness and thickness, > 2.8 screen (*r*  =  0.5), and TGW (*r*  =  0.2). TGW correlated positively with grain width (*r*  =  0.9), grain area (*r*  =  0.9) and negatively with several traits, including biomass, spike number, grain number and leaf temperature. The < 2.0 mm seed screen trait showed the strongest negative correlation with most traits. In addition, strong correlations also emerged between plant biomass and days to anthesis (*r*  =  0.8), leaf temperature and PH (*r*  =  0.4) and among SPAD, CCM200, leaf width and area (*r*  =  0.7–0.9). PCA clustering further highlighted distinct trait groupings (Figure 3b). The < 2.0 mm category was positioned opposite to TGW, SPAD, leaf area and seed dimension traits, whereas anthesis, PH and spike‐related traits formed a separate cluster. The relative correlation analysis showed that SPAD, LEF and flag leaf area maintained strong positive correlations with grain weight‐related traits under stress (data not shown).

### 3.3 Comparison of Heat‐Tolerant and Heat‐Sensitive Genotypes

Heat‐tolerant and heat‐sensitive genotypes were identified based on grain weight reduction. Genotypes with less than a 30% reduction in grain weight and a control grain weight exceeding 2.8 g/plant were classified as HD‐tolerant (Rose et al. 2022), whereas those with more than a 70% reduction were considered HD‐sensitive. From 345 genotypes, 33 HD‐tolerant and 20 HD‐sensitive lines were selected (Table S6).

**Figure 4:**
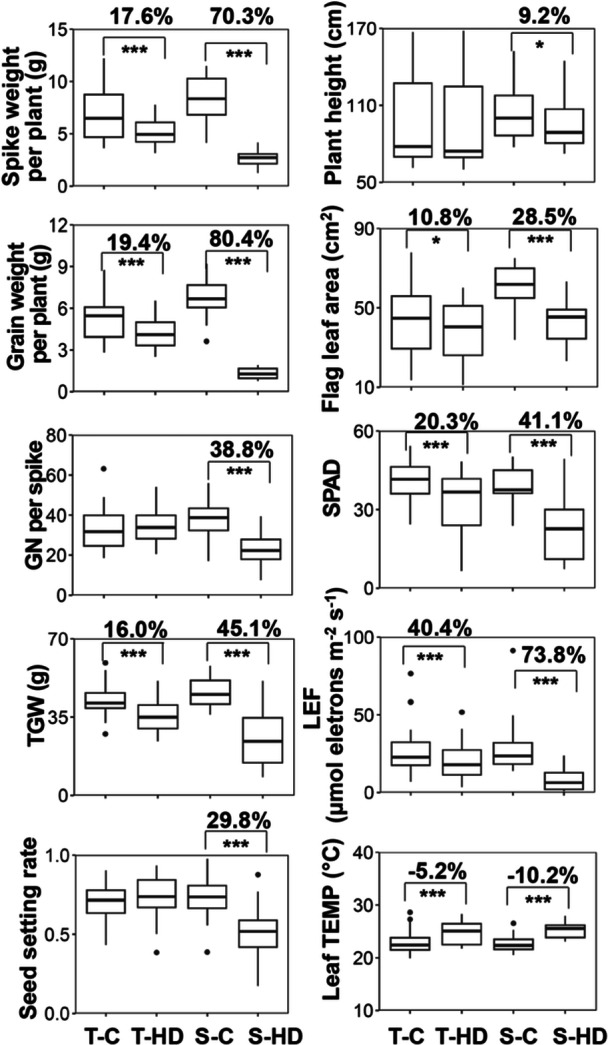
The main parameter differences on spike weight per plant, grain weight per plant, grain number (GN) per spike, thousand grain weight (TGW), seed‐setting rate, plant height, flag leaf area, SPAD, LEF and leaf temperature (TEMP) between HD‐tolerant and sensitive genotypes. T‐C, tolerant lines under control; T‐HD, tolerant lines under HD; S‐C, sensitive lines under control; S‐HD, sensitive lines under HD. *p < 0.05; ***p < 0.001.

Tolerant lines exhibited an average grain weight reduction of 19.4%, whereas sensitive lines showed an average reduction of 80.4%, reflecting similar trends in spike weight reduction (Figure 4). TGW decreased by 16% in the tolerant lines and by 45.1% in the sensitive ones. Grain number per spike, seed‐setting rate and PH remained largely unaffected in tolerant lines, while sensitive lines showed significant reductions of 38.8%, 29.8% and 9.2%, respectively. Sensitive lines also experienced more severe reductions in leaf area (18% higher), chlorophyll content (21% higher) and LEF (33% higher) with a doubled increase in leaf temperature compared to tolerant lines (Figure 4).

These findings underscore the importance of maintaining leaf functionality under HD stresses. Anthesis timing was found to influence HD response. Tolerant lines were divided into early (GH‐1T, < 90 days to anthesis) and late (GH‐2T, > 100 days to anthesis) flowering groups, with sensitive genotypes as a separate group (GH‐S). Among the 33 tolerant lines, 16 were early‐flowering and 17 were late‐flowering. Both tolerant groups maintained grain weight under HD, but late‐flowering lines experienced greater reductions in spike weight, chlorophyll content and LEF (Figure S5). Although the late‐flowering lines showed a slightly greater reduction in TGW (17.5% vs. 14.3%), they exhibited no significant reductions in PH, grain weight, grain number per spike, or flag leaf area. No significant treatment effects were observed within tolerant groups for seed‐setting rate, and leaf temperature was higher in late‐flowering lines across treatments.

Biplot analysis illustrated genotype performance. Under control conditions in the glasshouse, GH‐S overlapped with both tolerant groups, although GH‐1T showed marginally lower performance (Figure S6a). Under HD, distinct clustering emerged, clearly separating GH‐S from the tolerant groups (Figure S6b). Relative performance biplots (Figure S6c) showed complete separation of GH‐S, while GH‐1T and GH‐2T partially overlapped, indicating similar strong relative performance under stresses. Further strong positive correlations were observed between grain weight and spike weight per plant, grain number per spike, mini test weight, plant biomass and > 2.8 screen under both HD and control. In contrast, seed‐setting rate and LEF clustered with anthesis, PH and leaf temperature under control conditions (Figure S6a), but diverged under HD (Figure S6b), highlighting the specific impact of HD on seed‐setting and LEF.

### 3.4 Significant MTAs Identified Through GWAS

Phenotypic data analysis indicated that HD‐tolerant lines retained chlorophyll content and green leaf area. Therefore, from 47 traits (Table S3) under both control and HD treatments in the glasshouse, 19 key traits were selected for GWAS. These included CCM200, GWperPlant, main spike grain weight (MSSdwt), mean sub‐tiller grain weight (MeanTSdwt), leaf area (Leafarea), leaf width (Leafwidth), leaf length (Leaflength), LEF, PH, seed‐setting rate per plant (TotalSdSetting), main spike seed‐setting rate (MSSdsetting), mean sub‐tiller seed‐setting rate (MeanTSdSetting), mean grain number per spike (MeanSdNo), main spike grain number (MSSdNo), mean grain number per sub‐tiller spike (MeanTSdNo), SPAD, TGW per plant (TotalTGW), main spike TGW (MSTGW) and TGW on sub‐tiller spikes (TTGW). In addition, their HD/control ratios and anthesis were analysed, along with five key traits of days to anthesis, CCM200, GY, PH, TGW recorded in the 2023 Northam field trial (Figure S7).

**Figure 5:**
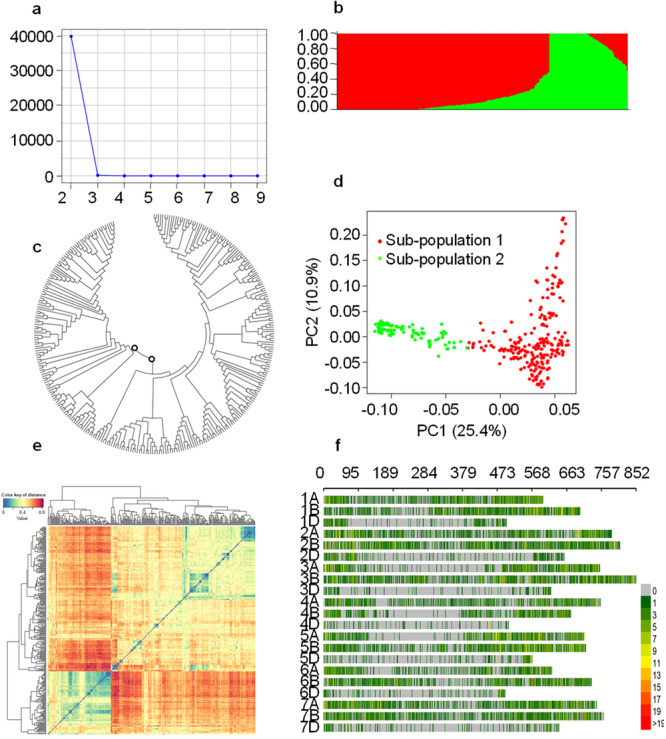
Population structure and kinship matrix of 319 accessions based on 15,429 SNPs and distribution of SNPs on 21 chromosomes. (a) Plot of delta *K* (2–9) and the presence of a peak at *K* = 2 hint at two subgroups; (b) population STRUCTURE analysis; (c) neighbour joining phylogenetic tree of 319 accessions; (d) plot of first principle component (PC1) against second principle component (PC2); (e) cluster heatmap of the population; (f) the assignment of SNPs to individual 21 chromosomes with unit of Mb on *x* axis. The number of SNPs within 1 Mb window size. [Color figure can be viewed at wileyonlinelibrary.com]

**Figure 6:**
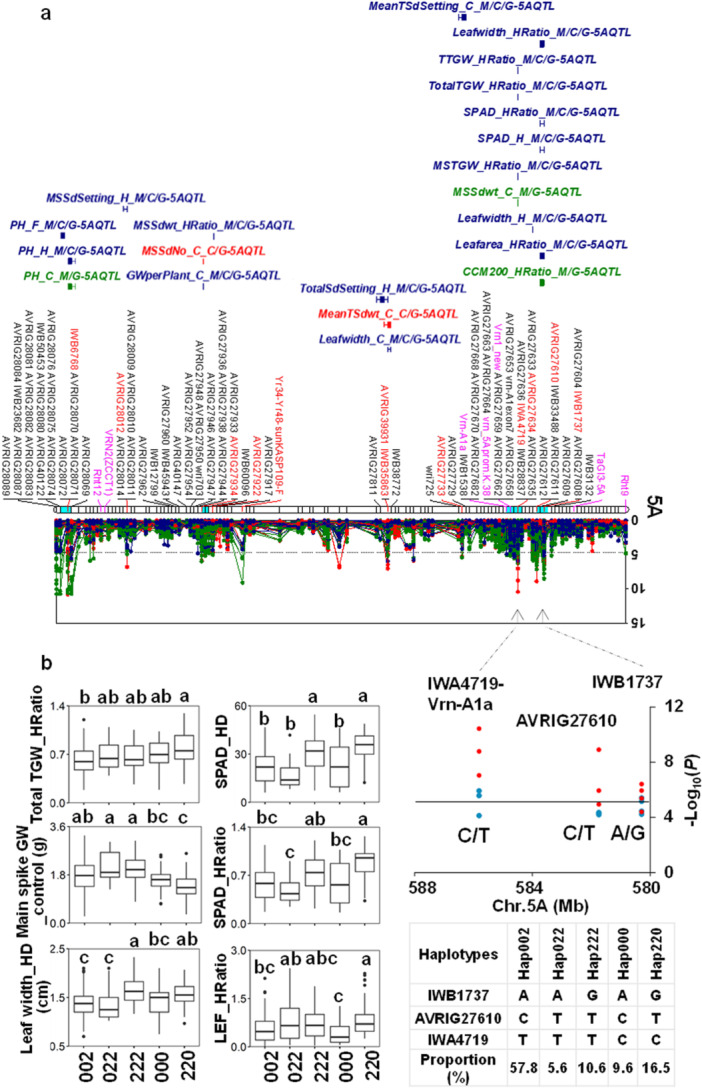
Significant 21 MTAs of grain weight per plant (GWperPlant), main spike (MSSdSetting) and sub‐tiller spike seed‐setting (MeanTSdSetting), SPAD and SPAD ratio (Hratio), leaf width and leaf area ratio, ratios of total TGW (TotalTGW) and sub‐tiller TGW (TTGW), main spike grain weight (MSSdwt) and grain number (MSSdNo) and plant height (PH) under HD (_H) and control (_C) on 5A (a), and (b) Local Manhattan plot (CPU in red; MLM in blue) and boxplots surrounding the cSNPs on 5A for the main spike grain weight (GW) under control; the ratio of total TGW, SPAD and LEF; SPAD value and leaf width under HD. M/C/G: MLM/CPU/GLM; values with the same letter in rows are not different at *p* = 0.05. [Color figure can be viewed at wileyonlinelibrary.com]

In summary, we used 19 traits under each of the two treatments in the glasshouse trial, along with one relative value per trait (19 traits × 3, plus anthesis), and five traits from the field, resulting in a total of 63 traits for GWAS. The heritability of these traits varied across two environments. Under control, 17/19 traits showed high broad‐sense heritability (*H* ^2^ > 0.7). Under HD, 9 traits remained highly heritable, most others were moderately heritable (0.54–0.69), except LEF (0.22). Among relative traits, CCM200, TotalTGW and MSTGW had *H* ^2^ > 0.5; TTGW, Leafwidth and TotalSdSetting were lower (0.39–0.44); several others had very low heritability ( < 0.1) (Table S3). From the STRUCTURE, PCA and cluster heatmap analysis, two subpopulations (*K* = 2) were identified (Figure 5a–e). SNP distribution across A, B and D genomes was 41.9%, 49.2% and 8.9%, respectively. Chromosome 3B had the highest coverage; 6D the lowest (Figure 5f). Three models (GLM, MLM, FarmCPU) were used in GWAS. In total, 124 cSNPs were identified across 51 traits on 19 chromosomes with 78 cSNPs associated with HD tolerance (Tables S7–S8, Figure 6, Figures S8–S22). These included 46 cSNPs associated with 15 traits under control (C), 43 from 19 traits under HD (H), 23 from 12 traits of HD ratio (R) and 12 from 4 field traits. The number of cSNPs per trait and chromosome is detailed as follows.

Twenty‐four cSNP blocks (within 3 Mb) exhibited pleiotropic effects and were associated with multiple traits (Table 1 and Table S9). For instance, AVRIG16378 on 1B was associated with four traits of grain number and seed‐setting under HD (Figure S9). AVRIG21318 on 2D was linked to leaf area and Leaf‐width HRatios (Figure S11). AVRIG21366 on 3 A was associated with SPAD traits (Figure S12). AVRIG22644 and AVRIG22646 on 3B were linked to grain number and seed‐setting under both HD and control environments (Figure S13), suggesting robust QTL. AVRIG40312 on 4D was linked to PH in all environments and located 2 Mb above *Rht2* (Figure S15). On chromosome 5A, 28 QTLs were identified (Table S8 and Figure 6). *Vrn1‐5A* (589.3 Mb) was associated with TGW and grain weight traits. Leaf‐related traits clustered near AVRIG27610 and IWB1737. PH loci were near *Rht12* (698.7 Mb) (Figure 6). On 5B, AVRIG28524 and AVRIG28526 were associated with LEF and sub‐tiller grain weight under HD. AVRIG29055 was 2.6 Mb from *Vrn1‐5B* (Figure S16). AVRIG29798 on 5D was 1.3 Mb above *Vrn1‐5D* and linked to TGW traits (Figure S17). Additional MTAs were found on 1A (Figures S8), 2B (Figures S10), 3D (Figures S12), 4A (Figures S14), 4B (Figures S15), 6A (Figures S18), 6B and 6D (Figure S19), 7 A (Figure S20) and 7B (Figure S21). For example, the high chlorophyll content was associated with marker wri756 on the short arm of 1A (CCM200), and grain weight of the sub‐tiller under HD was associated with AVRIG20303 on 2B (Figures S8 and S10). MTAs for flag leaf area and main spike grain weight under HD co‐localised on the long arm of 4A (Figure S14). Marker IWB56078 on 4B detected for PH was located 4 cM below the *Rht1* gene (Figure S15). A cSNP (AVRIG30272) associated with tiller TGW traits (TTGW_H, TTGW_Hratio) was detected on 6 A (Figure S18), whereas an MTA (AVRIG31032) for CCM200_F was detected on chromosome 6B and co‐localised with an anthesis‐related MTA (AVRIG39619) (Figure S19). AVRIG33074 (7A) was associated with multiple TGW traits (Figure S20). AVRIG34246 and IWB72576 (7B) were linked to field TGW and seed‐setting traits (Figure S21). The rest Manhattan plots generated with Farm‐CPU, MLM and GLM models on mainly the phenotypes under control were presented in Figure S22.

**Table 1:** Information of candidate SNPs (cSNPs) detected by GWAS.

Locus	Trait^a^	SNP ID	Chro. and cSNP block	LOD (CPU/MLM)	Effect (FarmCPU/MLM)	cSNP block interval (Mb)	Putative candidate genes
*QSdNo&Set _H‐1B*	MeanSdNo_H	**AVRIG16378**	1B‐1	5.2/3.2	−3.91/−3.34	552.4–558.4	TraesCS1B03G0883000/TraesCS1B03G0889300
	MeanTSdNo_H	AVRIG16378		8.5/4.5	−3.91/−4.04		
	MeanTSdSetting_H	AVRIG16378		5.9/4.1	−0.07/−0.06		
	TotalSdSetting_H	AVRIG16378		4.7/3.5	−0.04/−0.05		
*QLfA_H‐1B* b	Leafarea_H	**AVRIG16487**	1B	4.8/2.6	−2.46/−2.94		
*QLfA&W&L _Hratio‐2D*	Leafarea_HRatio	AVRIG21318	2D‐1	4/5.3	0.04/0.07	633.6–639.6	TraesCS2D03G1254100
	Leafwidth_HRatio	AVRIG21318		5.5/4.3	0.06/0.06		
	Leaflength_HRatio	AVRIG21328		5/4.6	0.01/0.01		
*QSPAD _H&Hratio‐3A*	SPAD_H	AVRIG21366	3A‐1	5.8/5.2	−2.17/−3.37	0–3.4	TraesCS3A03G0000100
	SPAD_HRatio	AVRIG21366		7.7/4.7	−0.07/−0.08		
*QSdSet&No _H&C‐3B*	MSSdSetting_H	**AVRIG22646**	3B‐1	4.1/4.6	0.04/0.07	22.6–28.6	TraesCS3B03G0082900/TraesCS3B03G0088700
	MSSdNo_H	AVRIG22644		5.7/5.6	2.95/4.37		
	MSSdNo_C	AVRIG22646		5.8/4	5.95/5.26		
	MSSdSetting_C	AVRIG22646		6.1/4.6	0.08/0.07		
	TotalSdSetting_C	AVRIG22646		4.8/3.5	0.06/0.06		
*QLfA&L _H&C‐3B*	Leafarea_H	**AVRIG22751**	3B‐2	7.1/4.4	5.15/5.67	61.2–67.2	TraesCS3B03G0203200
	Leafarea_C	AVRIG22751		6.7/4.4	5.98/7.01		
	Leaflength_H	AVRIG22751		6.6/4.9	2.42/3.37		
	Leaflength_C	AVRIG22751		5.5/4.8	2.38/3.3		
*QGW_H‐3B* b	GWperPlant_H	**AVRIG23283**	3B	6.3/4.2	0.36/0.52		
*QLfA _H&C‐4A*	Leafarea_H	AVRIG25551	4A‐1	8.1/4.6	−4.84/−5.42	748.7–754.7	TraesCS4A03G1245400
	Leafarea_C	AVRIG25551		8.7/4.9	−6.26/−6.87		
*QPH _C&H&F‐4D*	PH_C	AVRIG40312	4D‐1	9.7/5	8.38/8.65	13.6–19.6	TraesCS4D03G0067100 (*Rht‐D1*)
	PH_H	AVRIG40312		6.4/4.6	6.43/7.77		
	PH_F	AVRIG40312		5.8/5.6	2.69/4.21		
*QMSSdNo&Set _Hratio‐5A*	MSSdNo_HRatio	AVRIG26842	5A‐1	5.5/5.2	0.35/0.35	10.8–16.8	TraesCS5A03G0031000
	MSSdSetting _HRatio	AVRIG26842		10.5/6	0.28/0.27		
*QLfA&W&SPAD&LEF _H&Hratio‐5A*	CCM200_HRatio	**IWB1737**	5A‐2	4.4/4.2	0.06/0.09	577.4–585.2	TraesCS5A03G0905300/TraesCS5A03G0905400/TraesCS5A03G0912200/TraesCS5A03G0917300
	Leafwidth_HRatio	IWB1737		5.9/4.4	0.04/0.04		
	SPAD_H	IWB1737		6.4/5.4	2.56/3.89		
	SPAD_HRatio	IWB1737		5.4/5.2	0.06/0.09		
	Leafarea_HRatio	**AVRIG27610**		8.9/4.4	0.03/0.04		
	Leafwidth_H	AVRIG27610		5.9/4.2	0.09/0.08		
	LEF_HRatio	AVRIG27610		4.9/4.3	0.14/0.14		
*QMSSdwt&TGW _C&Hratio‐5A*	MSSdwt_C	AVRIG27634	5A‐3	0.3/4.1	0.03/0.19	583.9–590.3	TraesCS5A03G0935400 (*Vrn1‐5A*)/TraesCS5A03G0935500
	MSTGW_HRatio	**IWA4719‐Vrn‐A1a**		7/5.6	−0.07/−0.09		
	TotalTGW_HRatio	IWA4719		8.8/5.9	−0.08/−0.09		
	TTGW_HRatio	IWA4719		10.4/4.1	−0.09/−0.08		
*QTSdwt&LfW _C‐5A*	MeanTSdwt_C	AVRIG39931	5A‐4	6.5/3.7	−0.11/−0.12	618.5–624.5	TraesCS5A03G1030800/TraesCS5A03G1030900
	Leafwidth_C	IWB35863		7/4.3	0.07/0.08		
*QPH _C&F‐5A*	PH_C	Yr34‐Yr48‐sunKASP109‐F	5A‐5	0/4.1	−0.36/12.13	658.0–664.0	NA
	PH_F	Yr34‐Yr48‐sunKASP109‐F		0.5/4.3	1.65/5.61		
*QMSSdw&GW&MSSdNo _Hratio&C‐5A*	MSSdwt_HRatio	AVRIG27922	5A‐6	4.6/4.3	−0.13/−0.13	666.5–674.8	TraesCS5A03G1172800 (NAC)/TraesCS5A03G1172900/TraesCS5A03G1173100/TraesCS5A03G1173400
	GWperPlant_C	AVRIG27934		5.9/4.5	0.98/0.95		
	MSSdNo_C	AVRIG27934		5.1/3.9	5.22/4.88		
*QSdSet _H‐5A*	MSSdSetting_H	AVRIG28012	5A‐7	6.8/4	−0.04/−0.05	689.1–695.1	TraesCS5A03G1247500 (MYB)
	TotalSdSetting_H	**AVRIG28012**		6.7/4.2	‐0.04/−0.05		
*QPH _C&H&F‐5A*	PH_F	AVRIG28067	5A‐8	2.7/4.3	−2.21/−5.2	704.7–710.7	*Rht12*
	PH_C	**IWB6768**		8.2/6.4	−9.08/−13.44		
	PH_H	IWB6768		10.8/5.9	−9.68/−12.09		
*QLEF&TSdwt&CCM200 _H&C‐5B*	LEF_H	**AVRIG28524**	5B‐1	5.1/3.6	5.1/4.56	390.7–397.0	TraesCS5B03G0573000 (Myb)/TraesCS5B03G0578000/TraesCS5B03G0578100
	CCM200_C	AVRIG28526		6.5/4.1	2.14/2.72		
	MeanTSdwt_H	AVRIG28526		1.2/4.8	0.04/0.21		
*QCCM200 _H‐5B* b	CCM200_H	**AVRIG28989**	5B	6.2/3.6	1.54/1.97		
*QTSdwt _H‐5B* b	MeanTSdwt_H	**AVRIG29003**	5B	4.4/4.8	0.11/0.18		
*QF&LfL _F&H&C‐5B*	Anthesis_F	**AVRIG29028**	5B‐2	5.9/4.1	1.66/2.39	565.8–579.4	TraesCS5B03G0986000 (*Vrn1‐5B*)/TraesCS5B03G0986100 (MADS)
	Leaflength_C	AVRIG29055		4.5/3.3	−1.35/−1.79		
	Leaflength_H	AVRIG29055		4.9/3.3	−1.45/−1.82		
*QSPAD&SdSet _C‐5B*	SPAD_C	AVRIG29080	5B‐3	5.5/3.5	−2.78/−2.37	588.0–595.3	TraesCS5B03G1013000 (Myb)/TraesCS5B03G1019300 (NRT1)/TraesCS5B03G1023700 (NAC)/TraesCS5B03G1024100/TraesCS5B03G1024200
	TotalSdSetting_C	AVRIG29088		5.5/4	−0.05/−0.05		
	MeanTSdSetting_C	AVRIG29094		5.7/4.5	0.06/0.05		
*QGW _H‐5D* b	GWperPlant_H	**AVRIG29716**	5D	5.1/4.3	0.53/0.73		
*QF&TGW _C&Hratio‐5D*	Anthesis_C	IWB63558	5D‐1	5.4/2.9	3.56/4.03	462.9–472.5	TraesCS5D03G0894800 (*Vrn1‐5D*)/TraesCS5D03G0895000/TraesCS5D03G0888400/TraesCS5D03G0900500 (WRKY)
	MSTGW_HRatio	AVRIG29798		0.4/5	−0.03/−0.08		
	TotalTGW_HRatio	AVRIG29798		0.4/4.9	−0.03/−0.07		
*QLfW_H‐5D* b	Leafwidth_H	**AVRIG29853**	5D	4.9/3.7	−0.09/−0.08		
*QLfL_C&H‐6A*	Leaflength_C	AVRIG30239	6A‐1	5/2.2	−1.75/−2.08	93.0–99.0	TraesCS6A03G0284400
	Leaflength_H	AVRIG30239		5.3/2.1	−1.78/−2.05		
*QTTGW _H&Hratio‐6A*	TTGW_H	AVRIG30272	6A‐2	5.2/4.2	5.96/6.13	115.8–121.8	TraesCS6A03G0335200 (Auxin)
	TTGW_HRatio	AVRIG30272		9.8/5.2	0.15/0.17		
*QMSSdwt&MSTGW _HRatio&H‐7A*	MSSdwt_HRatio	AVRIG33074	7A‐1	5.9/5.6	0.3/0.3	158.0–164.0	TraesCS7A03G0464200
	MSTGW_HRatio	AVRIG33074		7.5/4.9	0.19/0.21		
	MSTGW_H	AVRIG33074		5/4.8	9.2/9.3		
*QTGW_H‐7A*	MSTGW_H	IWB27404	7A‐2	5.6/3.9	−3.43/−3.13	574.5–580.5	TraesCS7A03G0953300
	TotalTGW_H	IWB27404		6.1/4.5	−3.16/−2.97		
*QTGW&SdSet _F&H&C‐7B*	TGW_F	AVRIG34246	7B‐1	6.5/5.1	2.47/3.04	45.5–52.3	TraesCS7B03G0130600 (Ethylene)
	MeanTSdSetting_H	IWB72576		5.9/3.5	0.06/0.05		
	MSSdSetting_C	IWB72576		4.8/3.4	0.05/0.04		
	TotalSdSetting_C	IWB72576		5.2/4	0.04/0.04		

*Note*: SNP names in bold were used for haplotype analysis.

^a^ Acronyms refer to Table S2.

^b^ MTAs in the locus column were included in the haplotype analysis but not in the cSNP block.

### 3.5 Putative Candidate Genes and Favourable Haplotypes Controlling HD Tolerance

Notably, pleiotropic effects were observed in 24 cSNP blocks, each associated with multiple traits under similar or distinct conditions. For candidate gene identification, a 3 Mb genomic window upstream and downstream of each cSNP was defined as the cSNP block. Gene annotations were extracted from the Chinese Spring RefSeq v2.1, which was also used for SNP probe alignment. The size and gene count of each cSNP block are presented in Table S9. For example, in the 1B‐1 cSNP block (552.4–558.4 Mb), 23 genes were identified (Table S10). Among them, *TraesCS1B03G0883000* (a *MADS*‐box transcription factor) and *TraesCS1B03G0889300* (a glycosyl hydrolase involved in abiotic stress response) were proposed as key candidate genes. On 1B‐1, the SNP marker AVRIG16378 was repeatedly detected for grain number per spike and seed‐set under HD conditions, and was tightly linked to the leaf area MTA‐AVRIG16487. Haplotype analysis indicated that the Hap00 allele (AA) (with the largest proportion 67.3%), which is associated with significantly larger leaf area, contributed to higher average seed‐set and grain number per spike under HD stresses (Figure S9). This superior allele, Hap00, characterised by broader leaf area, is therefore desirable for improving HD tolerance.

In the 2D‐1 block (633.6–639.6 Mb), among 69 annotated genes, *TraesCS2D03G1254100*, encoding an auxin‐responsive protein, was identified as a likely candidate. This gene has also been previously linked to a GY QTL (El Gataa et al. 2021). On 3A‐1 (0–3.4 Mb), only 14 genes were annotated. Among them, *TraesCS3A03G0000100* (a cellulose synthase family protein) may contribute to SPAD phenotypes under HD stresse. In addition, four basic helix‐loop‐helix (bHLH) DNA‐binding proteins (TraesCS3A03G0002800, G0003000, G0003300, G0003900) and two subtilisin‐like proteases (TraesCS3A03G0004300 and G0004600), involved in gene regulation, stress signalling and protein turnover, cannot be excluded as potential candidates.

The 3B‐1 block (22.6–28.6 Mb) harbours two *MYB* transcription factor genes (*TraesCS3B03G0082900* and *G0088700*) likely involved in spike grain number and seed‐setting rate. In the 3B‐2 block (61.2–67.2 Mb), *TraesCS3B03G0203200*, a *WUSCHEL*‐related homeobox gene, may control leaf length and area under both stress and control conditions. On 3B‐1, MTAs for grain number and seed‐set (25.6 Mb) and 3B‐2 for leaf area and leaf length (64.2 Mb) were detected in both HD and control environments. An additional MTA for GWperPlant under HD (454.3 Mb) was identified further downstream. These three MTAs were subsequently analysed for their haplotype effects. Different haplotype combinations exhibited nearly identical patterns for leaf length and leaf area under both treatments, indicating that this locus shows no treatment‐specific effects on leaf area and that variation in leaf area is primarily driven by leaf length. The haplotype with the shortest leaf length, Hap202 (AGC), contributed to the highest grain weight per plant and the highest seed‐set rate under HD conditions (Figure S13).

In 4A‐1 (748.7–754.7 Mb), a *CONSTANS*‐like zinc finger protein gene (*TraesCS4A03G1245400*) may be associated with leaf area traits. On 4D‐1 (13.6–19.6 Mb), *Rht1‐4D* (*Rht2*) (18.8 Mb) was confirmed as a strong candidate for PH, consistent with prior QTL studies (Li et al. 2019; Govta et al. 2022).

Eight cSNP blocks were identified on chromosome 5A: (i) 5A‐1 (10.8–16.8 Mb): *TraesCS5A03G0031000*, a *SAUR*‐like auxin‐responsive gene, may affect grain number and setting ratio, (ii) 5A‐2 (577.4–585.2 Mb): Three auxin‐responsive genes (*G0905300*, *G0905400*, *G0917300*) and one *WRKY* transcription factor (*G0912200*) may regulate leaf width, area and chlorophyll content under HD stresses, (iii) 5A‐3 (583.9–590.3 Mb): Associated with TGW under HD and main spike grain weight under control. Likely candidates are two *MADS*‐box genes: *TraesCS5A03G0935400* (*Vrn1‐5A*) and *G0935500*, (iv) 5A‐4 (618.5–624.5 Mb): Two photosynthesis‐related genes (*TraesCS5A03G1030800*, *G1030900*) may underlie variation in sub‐tiller grain weight and leaf width, (v) 5A‐5: PH‐related cSNPs may be influenced by a transcription factor (*TraesCS5A03G1153400*) and a *NAC*‐domain gene (*G1153700*), (vi) 5A‐6 (666.5–674.8 Mb): Main grain weight ratio and grain weight may be regulated by four *NAC*‐domain genes, (vii) 5A‐7 (689.1–695.1 Mb): Candidate genes include *VRN2* (*ZCCT1*) located 3 Mb downstream (698.1 Mb) (Yan et al. 2004), and *TraesCS5A03G1247500*, a *MYB*‐related transcription factor, and (viii) 5A‐8 (704.7–710.7 Mb): Likely governed by *Rht12*, near SSR marker W5AC207‐5A (698.8 Mb) (Sun et al. 2019). Twelve QTLs were detected within the closely spaced 5A‐2 and 5A‐3 regions (580.4–587.3 Mb), where the *Vrn1‐5A* gene is located. Three common SNPs—IWB1737, AVRIG27610 and IWA4719 (*Vrn1‐5A*)—were used for haplotype analysis. The Hap222 haplotype, exhibiting significantly higher SPAD values, leaf width and LEF under HD, showed a high total TGW_HRatio and increased main‐spike grain weight under control conditions (Figure 6).

For chromosome 5B, following three cSNP blocks have been identified: (i) 5B‐1 (390.7–397.0 Mb): A *MYB* transcription factor (*TraesCS5B03G0573000*) and two *WAT1*‐related genes (*G0578000*, *G0578100*) may regulate sub‐tiller grain weight and LEF under HD stresses, (ii) 5B‐2 (565.8–579.4 Mb): Leaf length and flowering time (days to anthesis) could be influenced by *Vrn1‐5B* (*G0986000*) and another *MADS*‐box gene (*G0986100*), and (iii) 5B‐3 (588.0–595.3 Mb): Related to seed‐setting rate and SPAD under control. Candidate genes include *NAC* domain (*G1023700*, *G1024100*, *G1024200*), *NRT1*‐related (*G1019300*) and *MYB* transcription factor (*G1013000*). The cSNP blocks 5B‐1 and 5B‐2, located in the 393.7–576.4 Mb region, showed tight linkage and positive correlations among LEF, CCM200, sub‐tiller grain weight, and seed‐set under HD conditions, along with later anthesis and shorter leaf length. SNPs associated with sub‐tiller seed‐set rate (AVRIG28824), CCM200 (AVRIG28989), sub‐tiller grain weight (AVRIG29003) and anthesis (AVRIG29028, *Vrn1‐5B*) were used for haplotype analysis. The results indicated that the Hap2220 (AGCC) haplotype, which exhibited significantly higher CCM200 under HD and average days to anthesis, contributed to the highest grain weight and seed‐set per sub‐tiller under HD conditions (Figure S16).

On 5D‐1 (462.9–472.5 Mb), the primary candidate gene is *Vrn1‐5D* (*TraesCS5D03G0894800*), supported by another *MADS*‐box gene (*G0895000*) and a *WRKY* transcription factor (*G0900500*). A grain‐weight MTA on chromosome 5D (357.9 Mb) was linked to the cSNP block 5D‐1 (469.5 Mb), which contains *Vrn1‐5D*, and grain weight was positively correlated with days to anthesis. An additional MTA for leaf width (487.4 Mb) was identified further downstream. These three MTAs were used to assess superior haplotypes. The Hap220 haplotype showed the highest grain weight per plant under HD conditions, together with the longest duration to anthesis and increased leaf width under HD (Figure S17).

For chromosome 6A, there were following two blocks: (i) 6A‐1 (93.0–99.0 Mb): *TraesCS6A03G0284400*, a regulatory gene, may affect leaf length, and (ii) 6A‐2 (115.8–121.8 Mb): *TraesCS6A03G0335200*, a *SAUR*‐like auxin‐responsive gene, may contribute to TGW and TGW ratio under HD stresses. Similarly, on chromosome 7A, there were also two blocks: (i) 7A‐1 (158.0–164.0 Mb): *TraesCS7A03G0464200* (*E2F* transcription factor) may influence main spike TGW and TGW ratio. *TaGASR7‐A1* (170.6 Mb) 6 Mb downstream, known for its association with grain weight (Dong et al. 2014), may also play a role, and (ii) 7A‐2 (574.5–580.5 Mb): A potassium transporter (*TraesCS7A03G0953300*) may regulate TGW traits. *TaSAP1‐A1* (585.0 Mb), 4.5 Mb downstream, has been linked to TGW and grain number (Chang et al. 2013). For chromosome 7B, in 7B‐1 (454.8–523.3 Mb), an ethylene‐responsive transcription factor (*TraesCS7B03G0130600*) may contribute to TGW and seed‐setting traits under various treatments. *TaSAP1‐7B* (544.4 Mb) is another candidate based on proximity and functional evidence (Chang et al. 2013).

## 4 Discussion

### 4.1 Reduction in Grain Number on Sub‐Tiller Spikes Mainly Attributed to Low Seed‐Setting Rate

A significant positive correlation was observed between grain weight per plant and both grain number per spike and seed‐setting rate. Since the HD treatment began at anthesis of the main tiller, grain numbers decreased in both main and sub‐tiller spikes—with a 5.6% greater reduction in sub‐tiller spikes. This was primarily due to a 7.6% decrease in seed‐setting rate, accompanied by a 3.1% reduction in floret number. No significant change was observed for spikelets per spike in sub‐tiller spikes. Thus, the substantial loss in sub‐tiller spike grain number was driven mainly by reduced seed‐setting rate, followed by a decline in florets. In contrast, for main spikes, reduced floret number was the primary limiting factor, as seed‐setting rate remained relatively stable.

These differences between main and sub‐tiller spikes can be attributed to their respective developmental stages at the onset of HD stresses. Main spike florets were largely at pollination, whereas sub‐tiller spike florets were still developing. Five consecutive days of heat exposure without water disproportionately affected sub‐tiller florets, which faced combined HD stresses during flowering. This likely resulted in greater ovary and floret abortion, reducing seed‐setting rate (Guo et al. 2015). In contrast, main spike florets continued to flower and self‐pollinate successfully due to sufficient soil moisture for the first 2 days, which allowed evaporative cooling via transpiration. Notably, plants tolerate heat stress better when water is available (Mondal et al. 2023). Therefore, seed‐setting rate is more severely impacted when heat stress occurs before anthesis.

### 4.2 Traits Contributing to HD Tolerance

After HD stress screening, 33 genotypes were identified as HD tolerant. Among the 33 HD‐tolerant lines, several lines were sourced from Australian breeding programmes including AGT (e.g., Cutlass, Grenade CL Plus, Sting, Sunco, Sunprime, Ventura, H45) and LongReach Plant Breeders (e.g., LRPB Impala, Mustang, Oryx, Spitfire) and InterGrain (e.g., Rockstar, Westonia). Additional lines were obtained from VIDA Horsham (UK), China (e.g., HG 35, Yu series, x‐262), and the rest from the University of Tasmania, Launceston, Australia. Some of these have already been reported as drought‐tolerant or high‐yielding under low‐input conditions (Tausz‐Posch et al. 2015; Liu et al. 2022). Therefore, the current study provides further validation supporting their HD tolerance.

Five genotypes—LRPB Impala, Grenade CL Plus, LRPB Oryx, HG 35 and x‐262—stood out as superior HD‐tolerant lines due to stable grain weight per plant, chlorophyll recovery capacity and reasonable PH and flowering time. These genotypes can be considered promising candidates for future wheat breeding programmes aimed at enhancing the HD tolerance of wheat varieties. LRPB Impala exhibited the highest performance and demonstrated the greatest tolerance among the five genotypes while Egret is the most sensitive variety with the 91% reduction of grain weight per plant in the glasshouse. Compared to sensitive genotypes, HD‐tolerant lines showed smaller reductions in grain weight per plant, likely due to maintained grain number per plant and seed‐setting rate, as well as lower losses in TGW. Interestingly, these lines also exhibited only half the reduction in flag leaf area, chlorophyll content and LEF compared to sensitive ones—suggesting more sustained photosynthate contribution post‐stress. Biplot analyses showed strong relationships between leaf area, SPAD values, LEF, and grain weight, grain number, TGW and seed‐setting rate. In fact, 23 of the 33 tolerant lines had SPAD values above 20 after recovery, retaining over 50% of pre‐stress levels—indicating that greener leaves significantly aid grain filling under stress. These results are supported by previous studies linking stay‐green traits to improved grain size and yield under stress (Gous et al. 2016; Shirdelmoghanloo et al. 2022; Zhao et al. 2022; Chen et al. 2025). Mechanistically, stay‐green phenotypes involve increased photosynthetic efficiency and expression of key enzymes like Rubisco activase and soluble starch synthase (Spano 2003). However, breeders should avoid selecting stay‐green genotypes with poor yield potential under optimal conditions, as yield remains the most critical criterion (Ghanem and Al‐Farouk 2024). HD‐tolerant genotypes spanned a wide flowering window (74–147 days after sowing). Subsequent analyses showed significant differences among early‐ and late‐flowering groups in grain weight per plant, grain number, PH and flag leaf traits. Notably, late‐flowering genotypes maintained similar grain weight per plant despite experiencing sharper chlorophyll declines—suggesting compensation via stem WSC remobilisation.

### 4.3 Stable and Consistent MTAs

A total of 24 cSNP blocks were associated with multiple traits and/or identified across different environments. Among those blocks, 22 blocks overlapped with previously reported MTAs related to yield or stress tolerance. Therefore, these results validate previous findings, and the confirmed MTAs may prove useful in future wheat breeding programmes through marker‐assisted selection. For instance: (i) 3B‐1 overlapped with a previously reported drought QTL for GY (Bennett et al. 2012; Govta et al. 2022), matching grain number per spike and seed‐setting rate under HD here, (ii) 4D‐1 contained *Rht2*, repeatedly linked to PH QTLs (Li et al. 2019; Govta et al. 2022), (iii) MTAs for grain weight per spike and awn length were detected near the 5A‐1 cSNP block in late sowing and low rainfall trials, respectively (Devate et al. 2022; Govta et al. 2022). In addition, a QTL of the spike weight was about 2.8 Mb above the cSNP block (Bhatta et al. 2018), (iv) 5A‐2 showed consistency with QTLs for NDVI and spikelet fertility (Telfer et al. 2021; Devate et al. 2022), aligned with leaf area, leaf width, LEF and chlorophyll content (SPAD) identified in this study, (v) 5A‐3 (*Vrn1‐5A* region) matched MTAs for GY and grain number under well‐watered and heat trials (Bhatta et al. 2018; Devate et al. 2022), (vi) In addition, MTAs for plot yield in late sowing trials and effective spike number per plant under heat stress were identified on 5A‐7 (Li et al. 2019; Devate et al. 2022) which also coincides with associations for main spike and total seed‐setting rate under HD found in the present study. Previous research also indicated that the 5A‐7 region is associated with heat tolerance (Li et al. 2019), (vii) the 5B‐3 region was also suggested to contribute to heat tolerance (Li et al. 2019), corresponding with traits like grain weight, seed‐setting rate, grain number per plant, spikelet fertility and chlorophyll content under heat or low rainfall (Bhatta et al. 2018; Telfer et al. 2021; Devate et al. 2022), (viii) The region of 5D‐1 was linked to anthesis, TGW ratio, spikelet fertility and NDVI (Telfer et al. 2021; Devate et al. 2022), (ix) 6A‐1 block corresponded to GY per plant under heat, drought and non‐stress conditions (Li et al. 2019). Other overlapping MTAs included effective spike number, awn length, harvest index and NDVI across both irrigated and restricted irrigation environments. The QTL on cSNP regions of 6A‐2 and 7A‐2 was not reported in those studies. In summary, our study confirmed several previously reported MTAs and identified novel MTAs for the first time.

### 4.4 Tight Chromosome Linkage Between Stay‐Green Traits and Yield Components

Phenotypic analysis revealed that HD‐tolerant lines experienced less reduction in chlorophyll content and flag leaf area. GWAS results revealed strong linkages between cSNPs associated with SPAD values (CCM200), leaf area, seed‐setting rate and grain weight components, such as grain number, and TGW under HD conditions. On chromosome 1B, the cSNP block for seed‐setting rate and grain number under HD was located 31.4 Mb upstream of the cSNP linked to leaf area. Further haplotype analysis showed that the Hap00 exhibited a large leaf area under HD, which contributed to the high seed‐setting and grain number per spike under HD. The largest proportion of the haplotype indicates the important loci selection during wheat breeding. Notably, the *TaFT3‐B1* gene (588.5 Mb) on 1B, acting as a flowering promoter, was 1.6 Mb downstream of the leaf area‐associated cSNP, suggesting its potential contribution to the observed phenotype as flowering genes influence plant architecture and development, including flag leaf area (Amo et al. 2022).

On chromosome 5A, further haplotype analysis demonstrated the Hap222 with greater SPAD values and LEF, increased leaf width under HD, showed a high total TGW_HRatio and increased main‐spike grain weight under control conditions. A QTL for chlorophyll content was consistently identified in the same region (582.4 Mb) on 5A under dryland conditions, further indicating the importance (Yang et al. 2022). Likewise, the haplotype analysis on the blocks 5B‐1 and ‐2 revealed that the Hap2220 with higher chlorophyll content under HD contributed to the highest grain weight under HD conditions. Hap2220 associated with early anthesis holds value for HD‐tolerant breeding. Similar co‐localisations were noted on 5D, the Hap220 haplotype exhibited the highest grain weight per plant under HD conditions, along with the greater leaf width under HD. Hap220, harbouring a late anthesis allele, indicated the wild‐type of *Vrn1‐5D* exhibited broad leaf and contributed to the high GY under HD conditions. Different from above, the leaf area, mainly controlled by the leaf length on 3B‐2, was not affected by the HD treatment. The shorter leaf length haplotype‐Hap202 contributed to the grain weight per plant under HD. The Hap202 is suggested to be used for HD wheat breeding.

In summary, the results above, from the molecular level, further demonstrate that the significant correlations between the HD tolerance and high levels of leaf width, leaf area and chlorophyll content under HD conditions. Similar findings have been reported in recently published studies (Chen et al. 2020; Li et al. 2023; Du et al. 2024; Abbas et al. 2025). In general, tolerant varieties that maintain green leaf area likely exhibit better cellular membrane stability, more effective osmotic adjustment and regulated antioxidant production like anthocyanins, which together protect chloroplasts from HD‐induced damage, and allow plants to retain greater photosynthetic capacity and water‐cooling potential. A high chlorophyll content further indicates a functional photosystem II (PSII) and an efficient electron transport chain (Bheemanahalli et al. 2022; Chen et al. 2025).

An exception was observed for reduced leaf area, which was influenced by leaf length rather than by the HD treatment. In fact, the haplotype on chromosome 3B with shorter leaf length contributed to improved HD tolerance. Shorter leaf length, which reduces surface area, can directly decrease the water loss through transpiration. With less exposure to solar radiation, shorter leaf length can reduce the heat load, thus low canopy temperatures. Therefore, shorter leaf length can mitigate the HD stresses. Previous studies have shown that smaller leaf area is adaptive under water‐limited conditions (Yang et al. 2016; Wang et al. 2019). The Hap202 haplotype on 3B, which confers shorter leaf length and leads to higher grain weight under HD, may therefore be valuable for breeding wheat with enhanced HD tolerance.

## 5 Conclusions

In this study, a 5‐day HD stress treatment during anthesis was applied to 345 wheat genotypes in a controlled glasshouse setting as well as a late‐sowing field experiment. Major phenotypic reductions due to HD‐stresses were observed in the glasshouse across key traits, including chlorophyll content, leaf area, biomass, spikelet and floret number, seed‐setting rate, TGW and grain size metrics. However, the HD‐tolerant lines exhibited minimal grain weight loss and maintained significantly higher levels of chlorophyll and green leaf area compared to sensitive lines. This resilience was further supported by GWAS findings, which revealed tight genetic linkages between SPAD, flag leaf area and leaf width and GY components. The superior haplotypes identified in this study can be used in a wheat breeding programme aimed at enhancing HD tolerance. For instance, Hap00 on 1B, Hap202 on 3B, Hap222 on 5A, Hap2220 on 5B and Hap220 on 5D, as well as the five superior HD‐tolerant genotypes—LRPB Impala, Grenade CL Plus, LRPB Oryx, HG 35 and x‐262 could prove useful in future wheat breeding programmes aimed at enhancing HD‐tolerance.

In summary, the study suggests that smaller reductions in SPAD and flag leaf area are reliable early indicators of HD tolerance. The stability of leaf width and chlorophyll content is particularly valuable screening traits. Furthermore, identified superior haplotypes, cSNPs and candidate genes provide strong targets for further research and breeding. Finally, the selected genotypes offer valuable resources for wheat breeding programmes focused on HD tolerance improvement.

## Ethics Statement

The authors have nothing to report.

## Conflicts of Interest

Author Rajeev K. Varshney and some co‐authors may have prior or ongoing scientific collaborations and publications with the Editor‐in‐Chief and/or members of the journal's editorial board; however, these relationships had no influence on the editorial handling of this manuscript. All editorial decisions, including the selection of the Handling Editor and peer reviewers, were made independently by the journal. The authors had no involvement in these processes and were not aware of the identities of the reviewers at any stage of peer review.

## Supporting information

Supporting File 1

Supporting File 2
