# Supplementary material for: Genomic and Physiological Insights Into Heat–Drought Tolerance in Wheat Through GWAS and Phenotypic Evaluation
Source: Plant Cell Environ. 2026 Apr 22;49(8):5511–29. doi: 10.1111/pce.70510 (PMC13353619; doi:10.1111/pce.70510)
Supplement: Supplementary file 1 — Supporting File 1 [file PCE-49-5511-s002.pptx]

## Slide 1
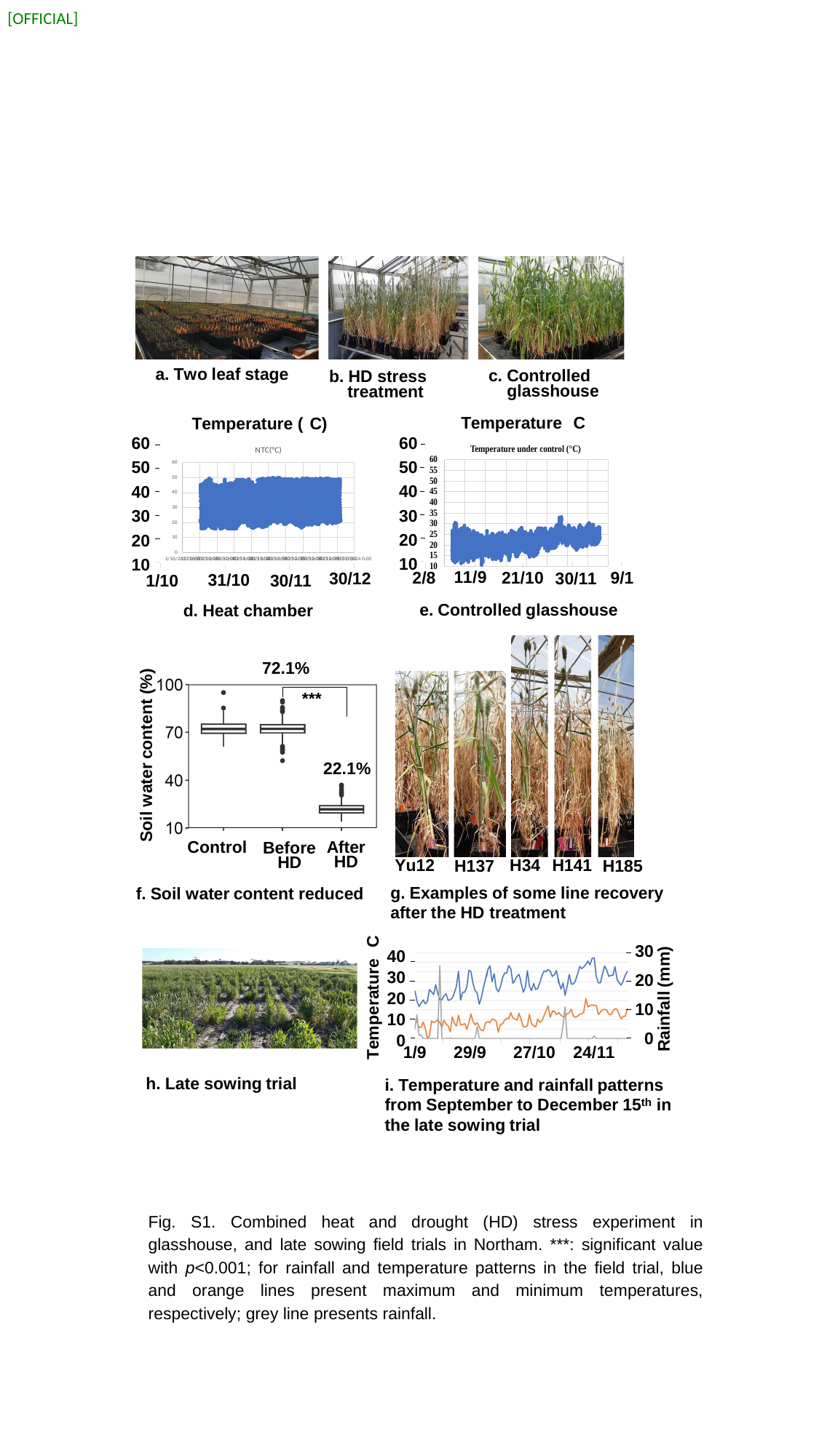

Fig. S1. Combined heat and drought (HD) stress experiment in glasshouse, and late sowing field trials in Northam. ***: significant value with p<0.001; for rainfall and temperature patterns in the field trial, blue and orange lines present maximum and minimum temperatures, respectively; grey line presents rainfall.

## Slide 2
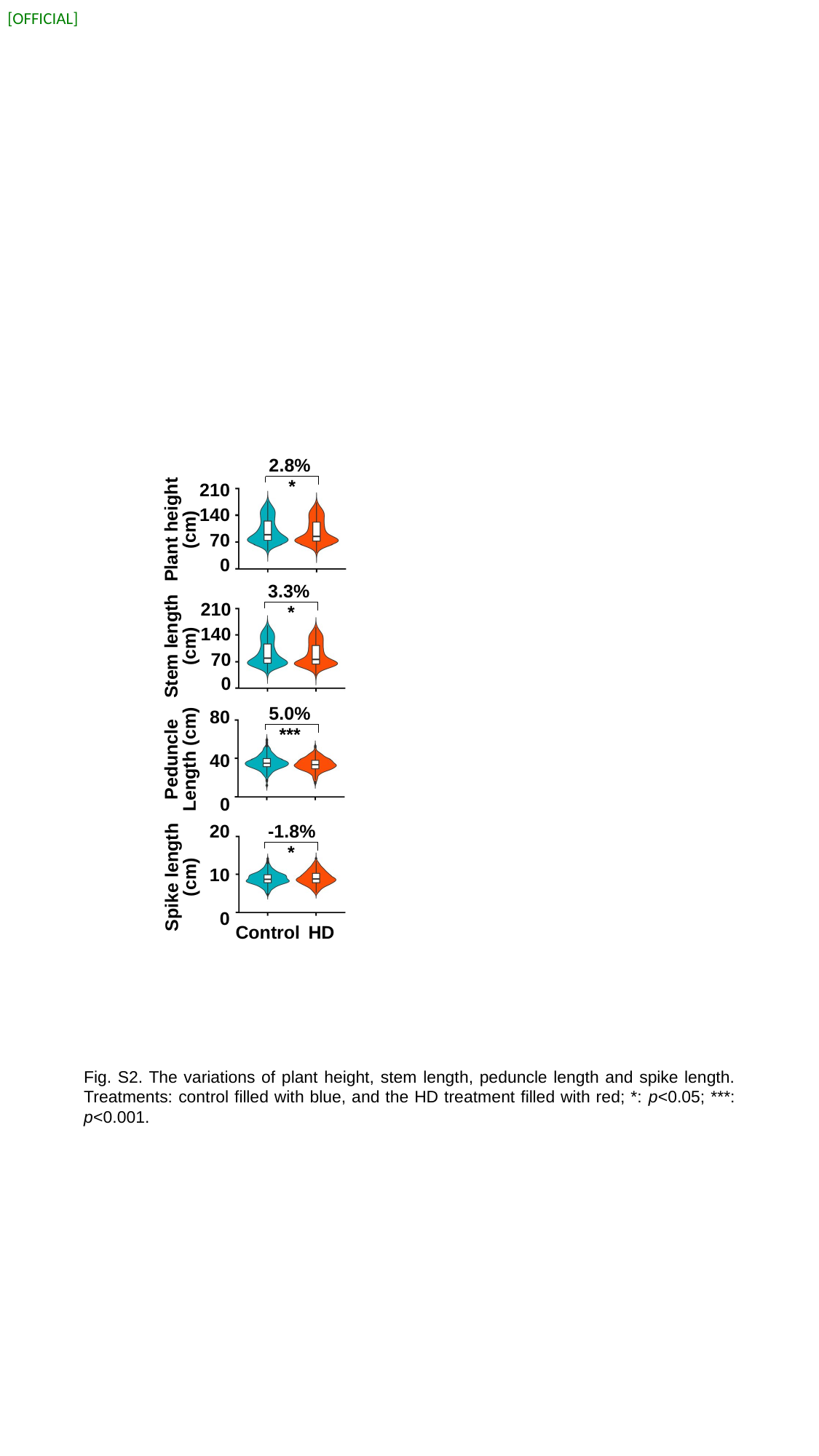

Fig. S2. The variations of plant height, stem length, peduncle length and spike length. Treatments: control filled with blue, and the HD treatment filled with red; *: p<0.05; ***: p<0.001.

## Slide 3
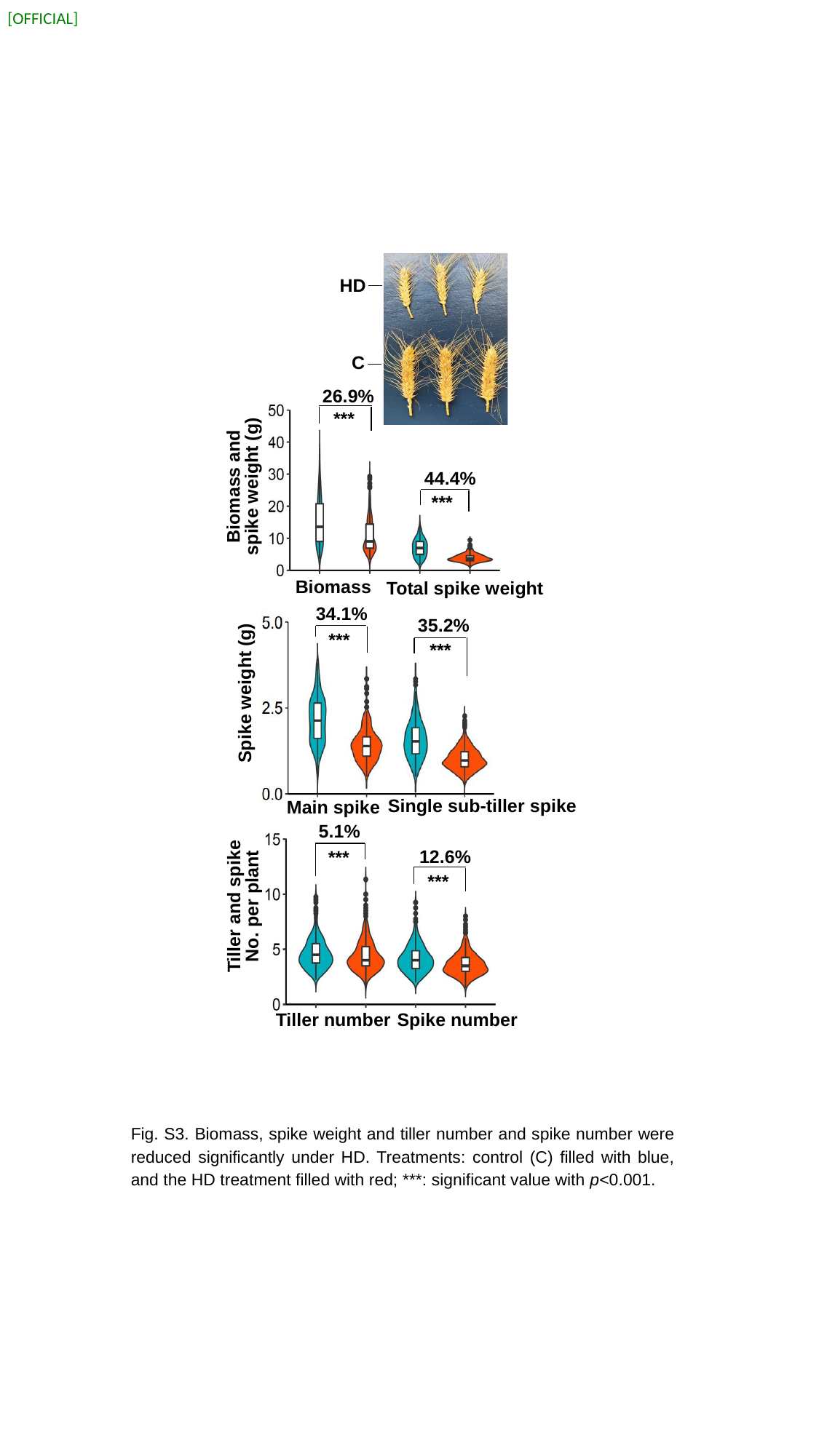

Fig. S3. Biomass, spike weight and tiller number and spike number were reduced significantly under HD. Treatments: control (C) filled with blue, and the HD treatment filled with red; ***: significant value with p<0.001.

## Slide 4
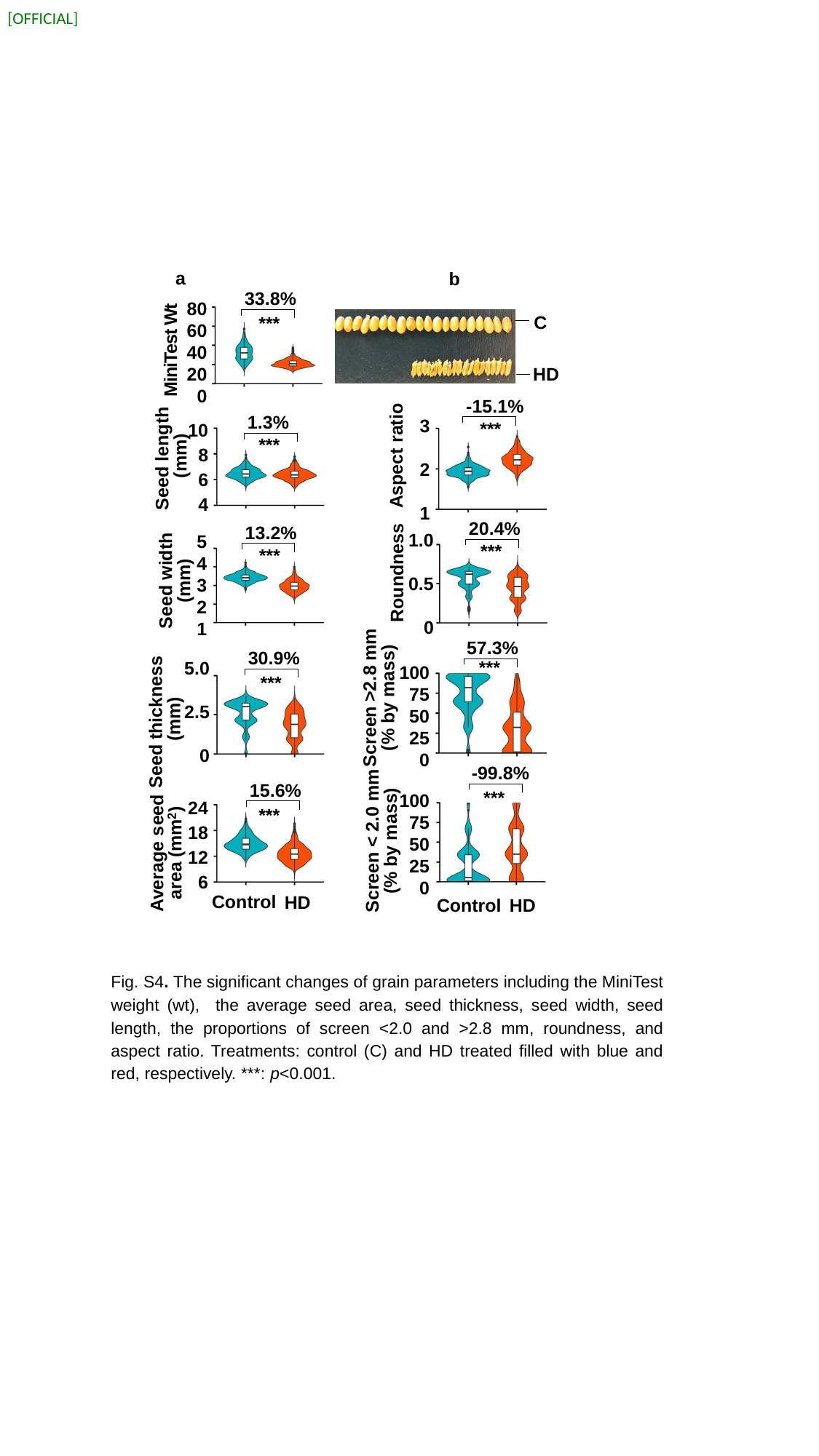

Fig. S4. The significant changes of grain parameters including the MiniTest weight (wt), the average seed area, seed thickness, seed width, seed length, the proportions of screen <2.0 and >2.8 mm, roundness, and aspect ratio. Treatments: control (C) and HD treated filled with blue and red, respectively. ***: p<0.001.

## Slide 5
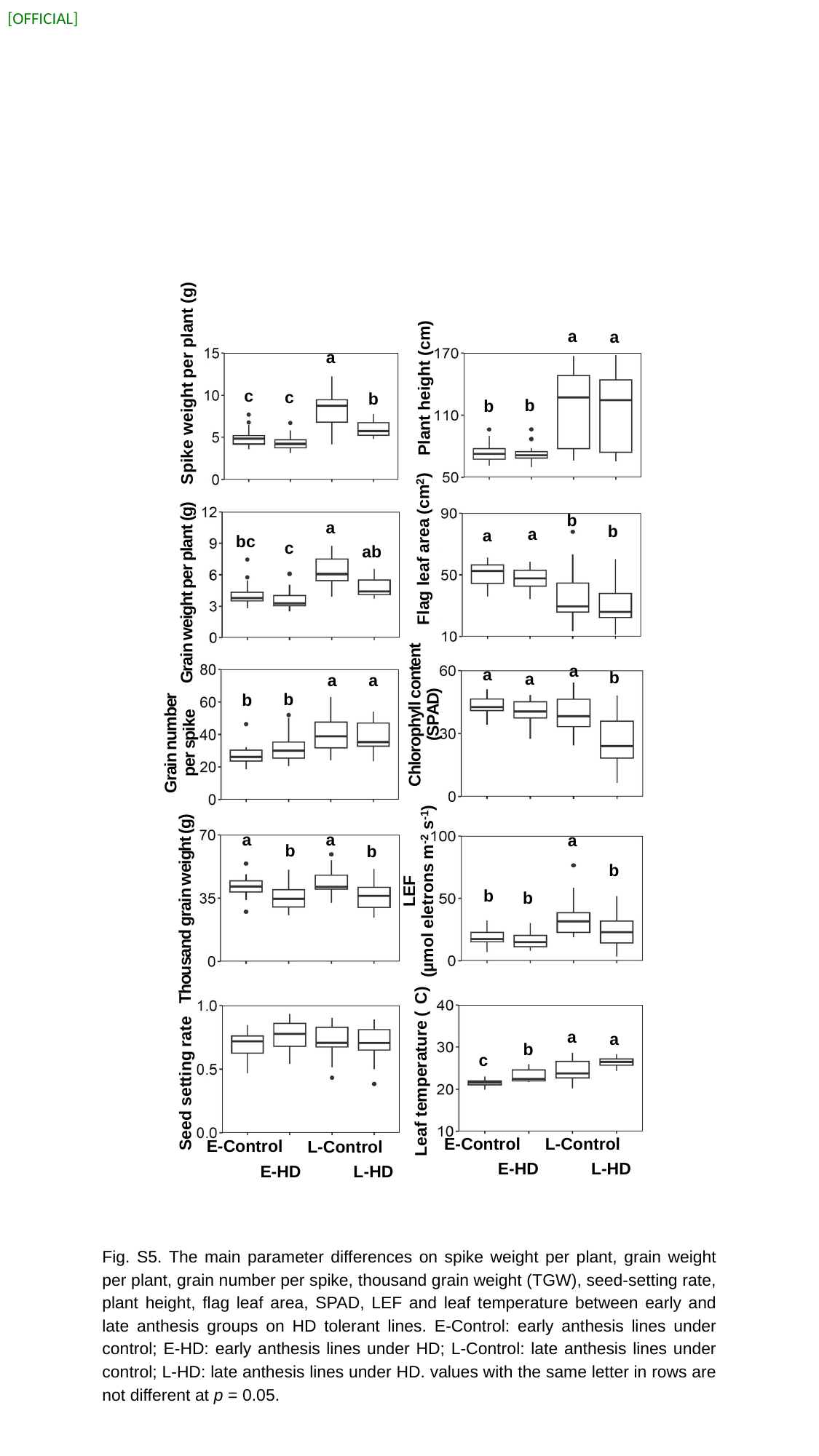

Fig. S5. The main parameter differences on spike weight per plant, grain weight per plant, grain number per spike, thousand grain weight (TGW), seed-setting rate, plant height, flag leaf area, SPAD, LEF and leaf temperature between early and late anthesis groups on HD tolerant lines. E-Control: early anthesis lines under control; E-HD: early anthesis lines under HD; L-Control: late anthesis lines under control; L-HD: late anthesis lines under HD. values with the same letter in rows are not different at p = 0.05.

## Slide 6
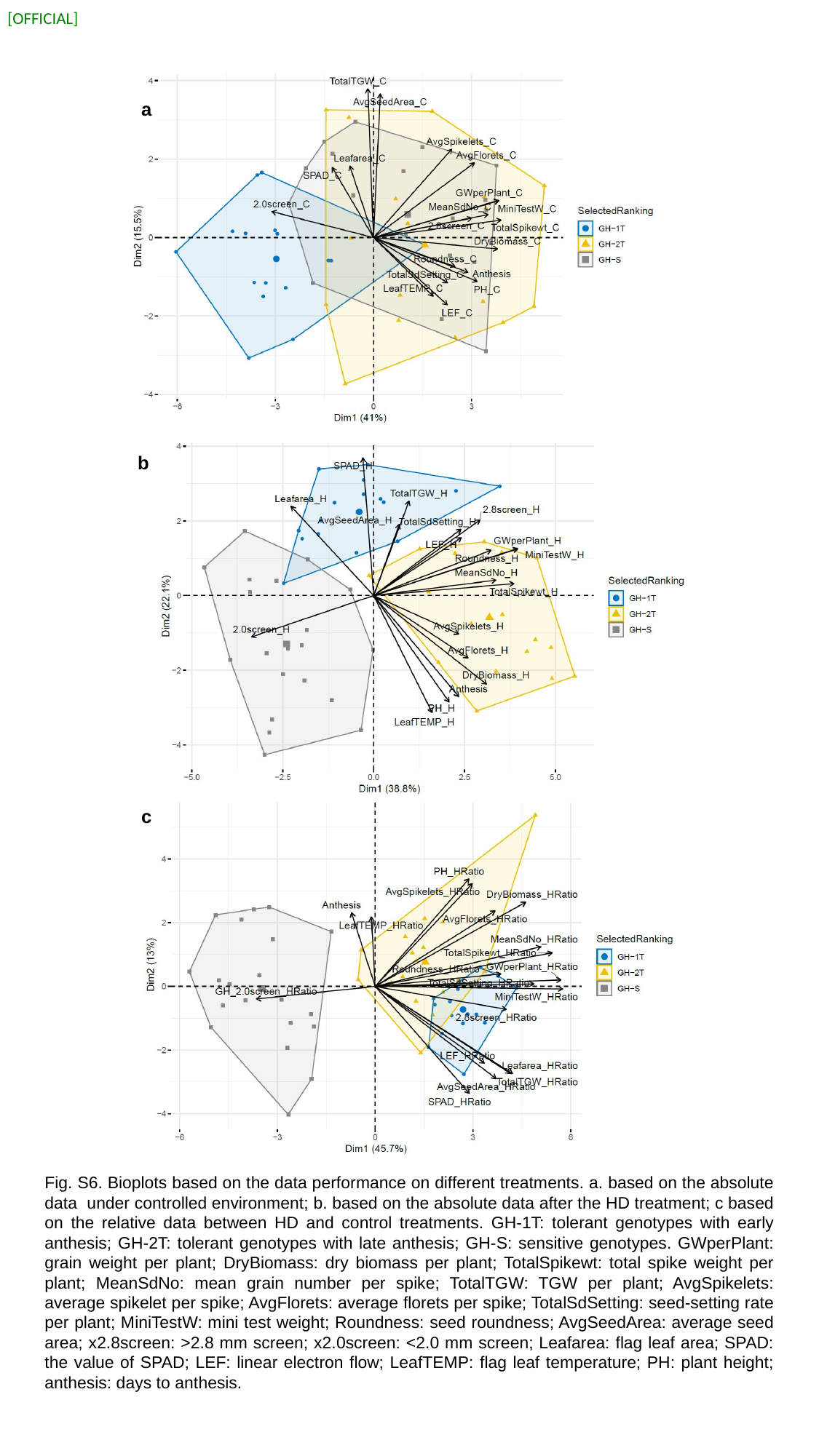

Fig. S6. Bioplots based on the data performance on different treatments. a. based on the absolute data under controlled environment; b. based on the absolute data after the HD treatment; c based on the relative data between HD and control treatments. GH-1T: tolerant genotypes with early anthesis; GH-2T: tolerant genotypes with late anthesis; GH-S: sensitive genotypes. GWperPlant: grain weight per plant; DryBiomass: dry biomass per plant; TotalSpikewt: total spike weight per plant; MeanSdNo: mean grain number per spike; TotalTGW: TGW per plant; AvgSpikelets: average spikelet per spike; AvgFlorets: average florets per spike; TotalSdSetting: seed-setting rate per plant; MiniTestW: mini test weight; Roundness: seed roundness; AvgSeedArea: average seed area; x2.8screen: >2.8 mm screen; x2.0screen: <2.0 mm screen; Leafarea: flag leaf area; SPAD: the value of SPAD; LEF: linear electron flow; LeafTEMP: flag leaf temperature; PH: plant height; anthesis: days to anthesis.

## Slide 7
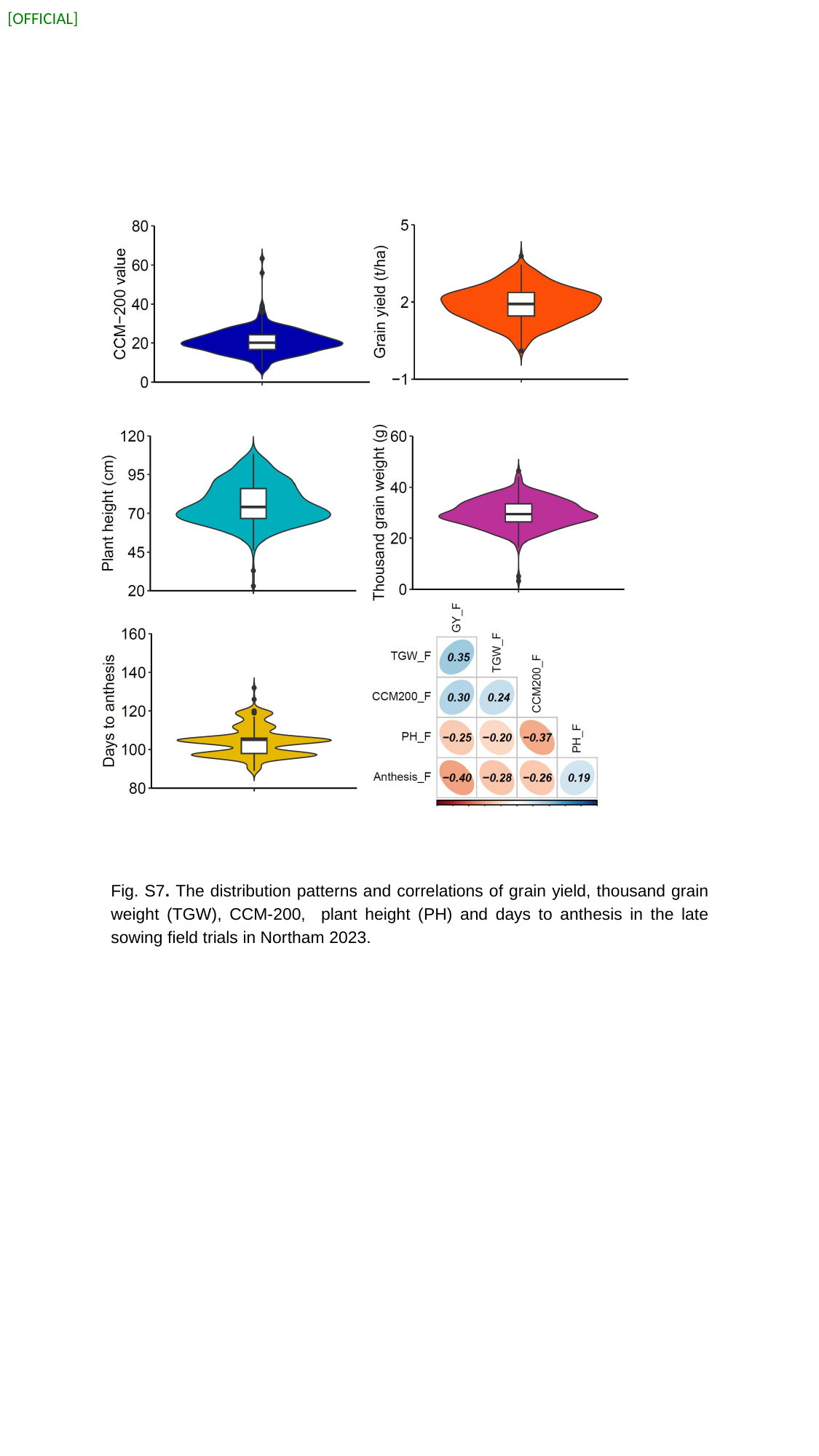

Fig. S7. The distribution patterns and correlations of grain yield, thousand grain weight (TGW), CCM-200, plant height (PH) and days to anthesis in the late sowing field trials in Northam 2023.

## Slide 8
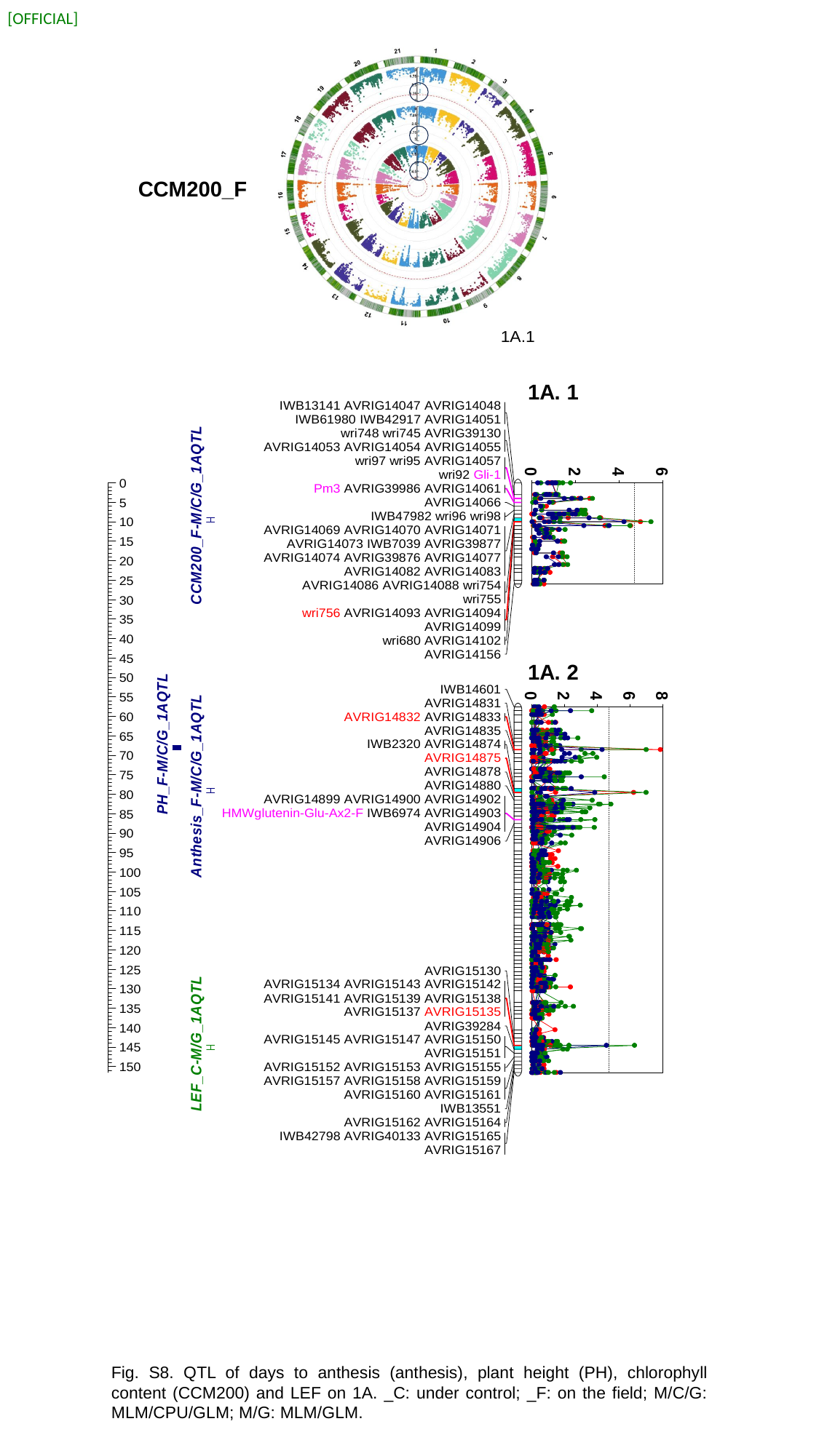

Fig. S8. QTL of days to anthesis (anthesis), plant height (PH), chlorophyll content (CCM200) and LEF on 1A. _C: under control; _F: on the field; M/C/G: MLM/CPU/GLM; M/G: MLM/GLM.

## Slide 9
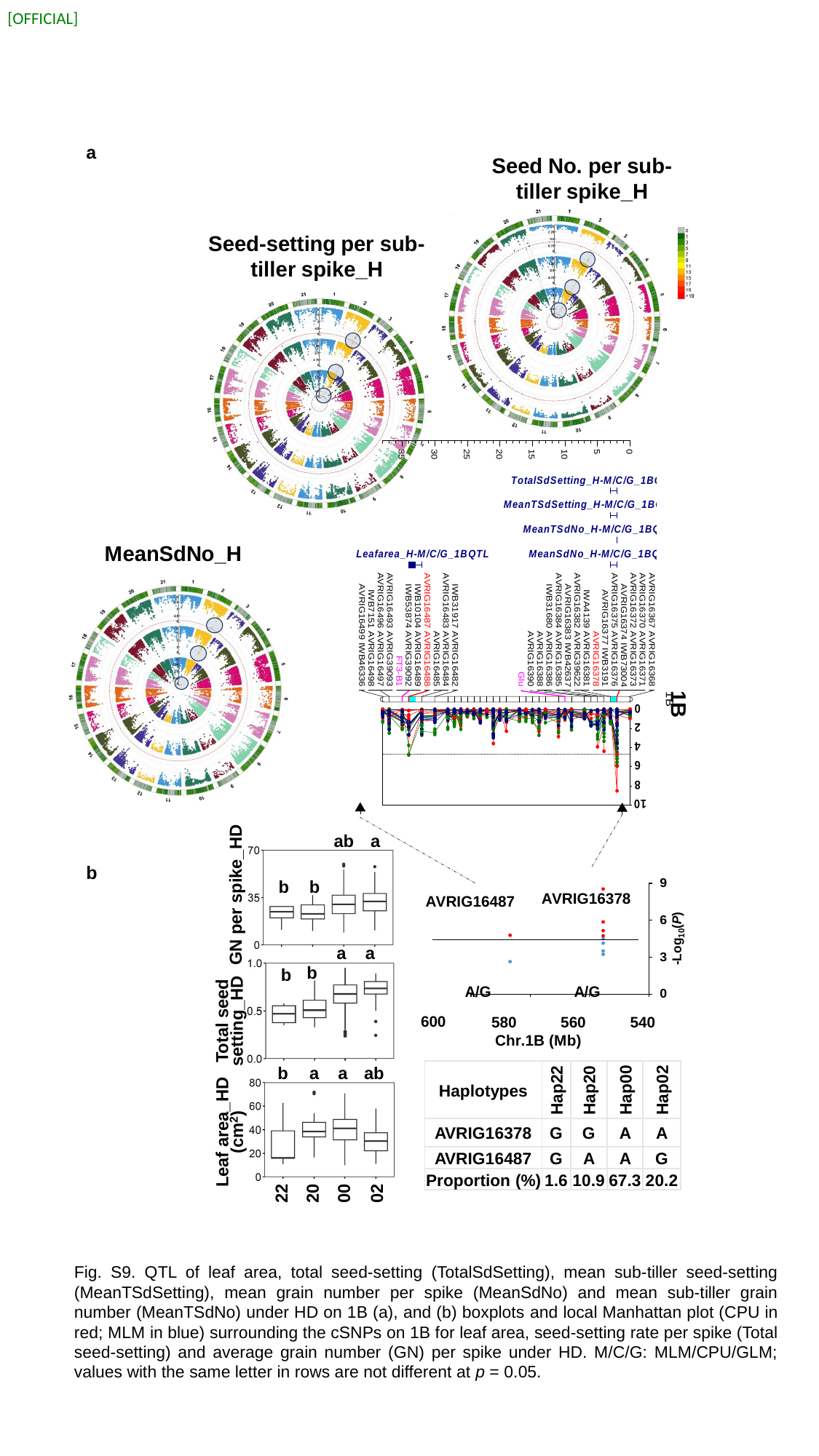

Fig. S9. QTL of leaf area, total seed-setting (TotalSdSetting), mean sub-tiller seed-setting (MeanTSdSetting), mean grain number per spike (MeanSdNo) and mean sub-tiller grain number (MeanTSdNo) under HD on 1B (a), and (b) boxplots and local Manhattan plot (CPU in red; MLM in blue) surrounding the cSNPs on 1B for leaf area, seed-setting rate per spike (Total seed-setting) and average grain number (GN) per spike under HD. M/C/G: MLM/CPU/GLM; values with the same letter in rows are not different at p = 0.05.

## Slide 10
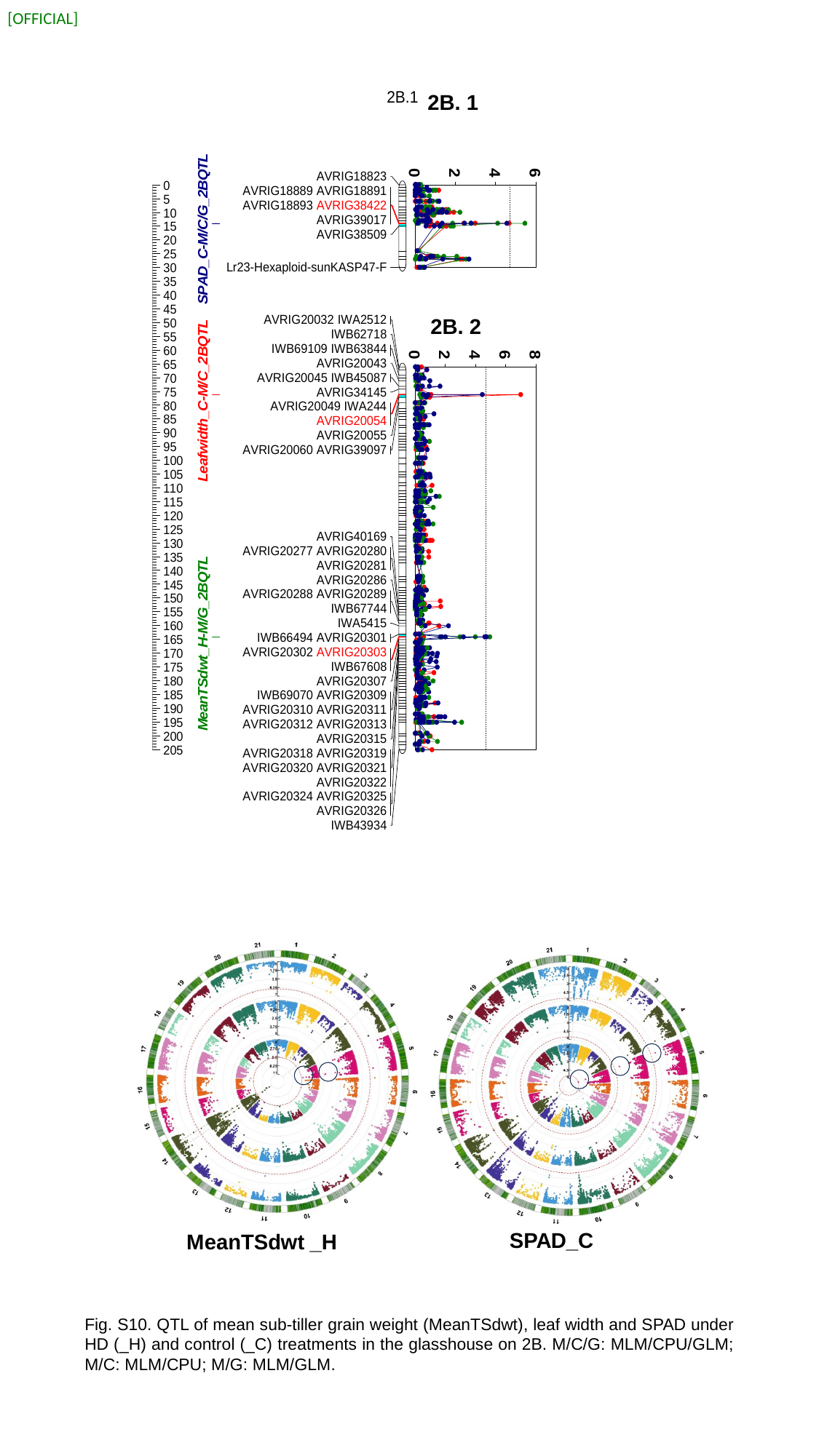

Fig. S10. QTL of mean sub-tiller grain weight (MeanTSdwt), leaf width and SPAD under HD (_H) and control (_C) treatments in the glasshouse on 2B. M/C/G: MLM/CPU/GLM; M/C: MLM/CPU; M/G: MLM/GLM.

## Slide 11
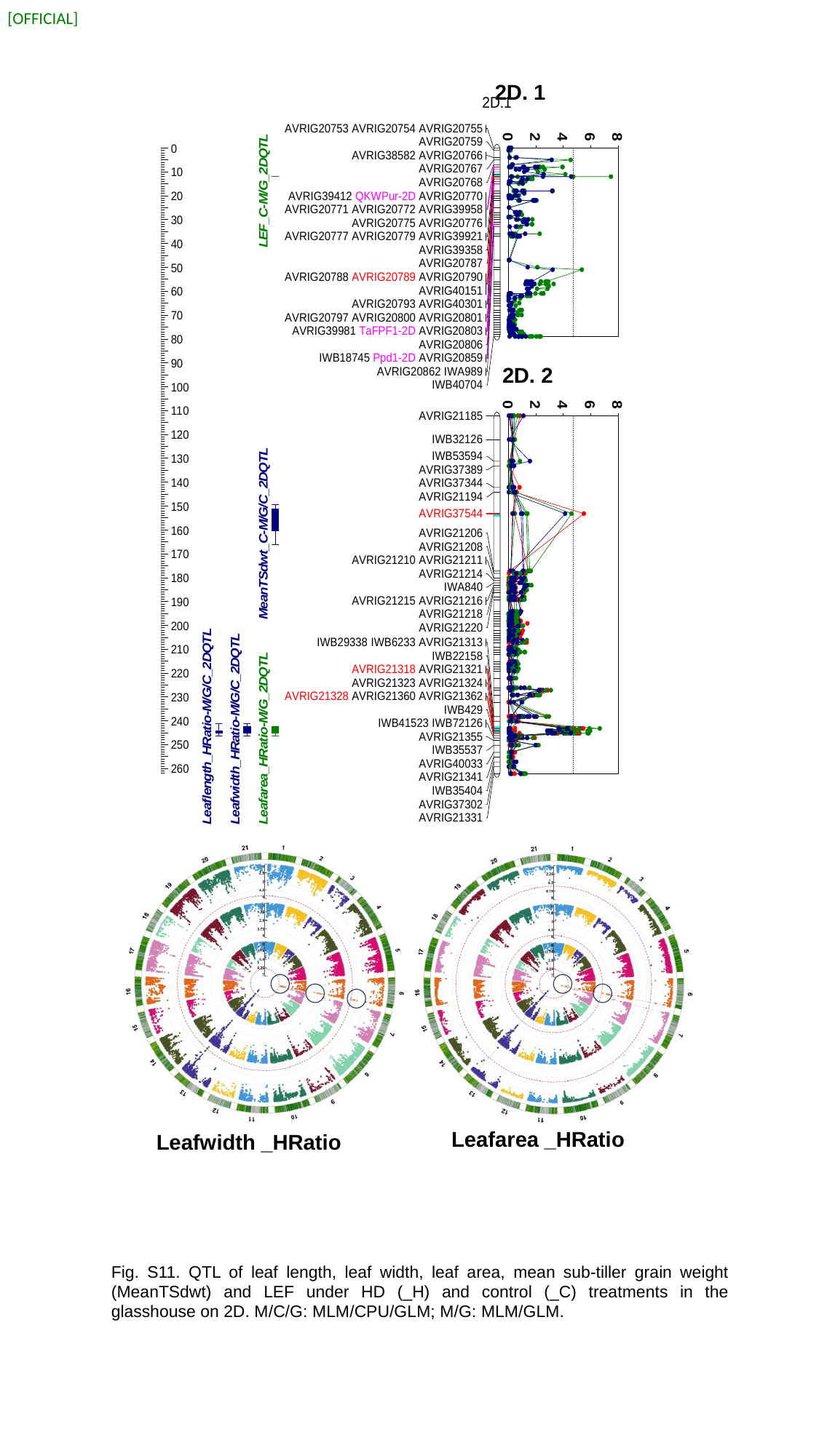

Fig. S11. QTL of leaf length, leaf width, leaf area, mean sub-tiller grain weight (MeanTSdwt) and LEF under HD (_H) and control (_C) treatments in the glasshouse on 2D. M/C/G: MLM/CPU/GLM; M/G: MLM/GLM.

## Slide 12
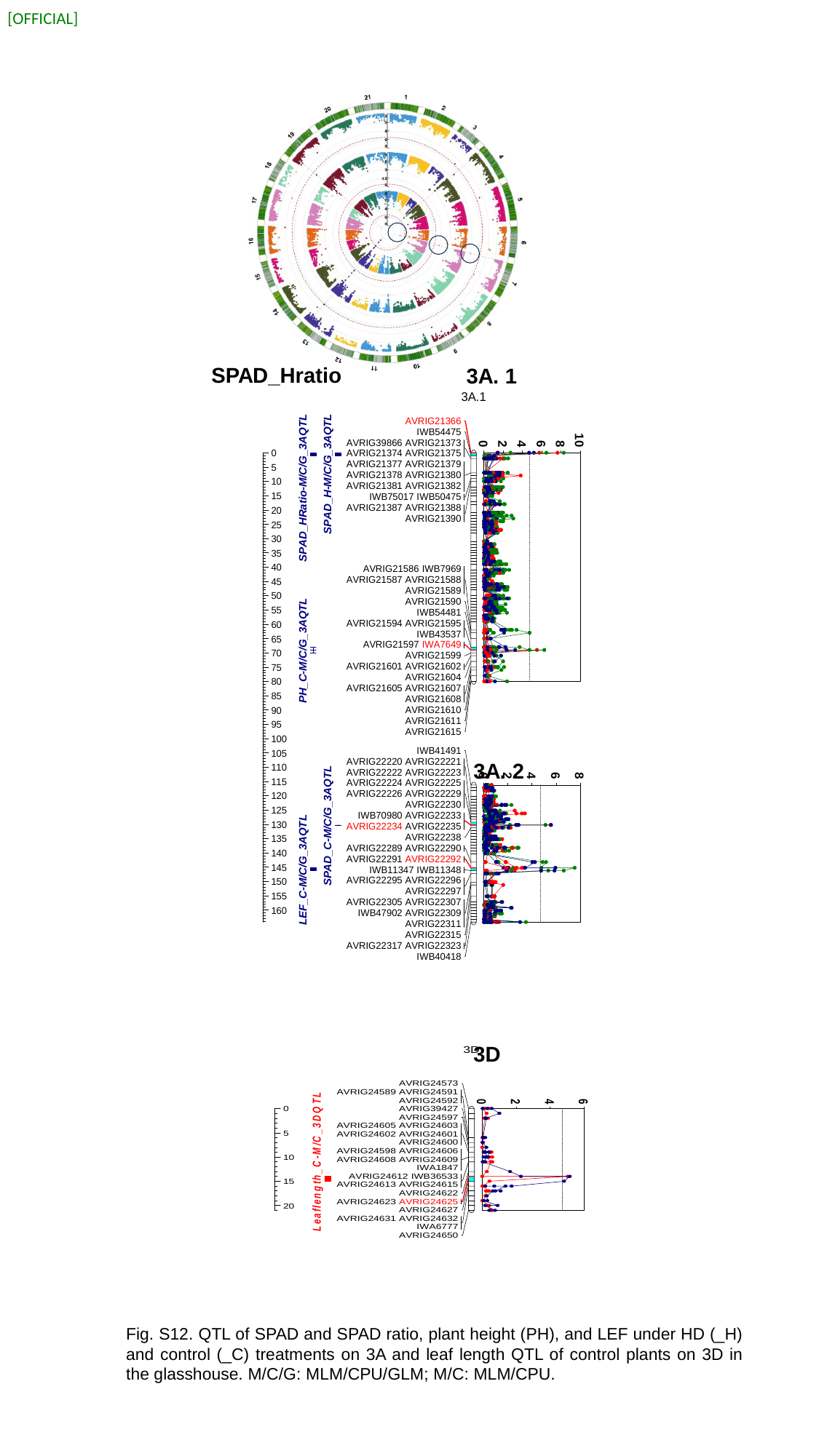

Fig. S12. QTL of SPAD and SPAD ratio, plant height (PH), and LEF under HD (_H) and control (_C) treatments on 3A and leaf length QTL of control plants on 3D in the glasshouse. M/C/G: MLM/CPU/GLM; M/C: MLM/CPU.

## Slide 13
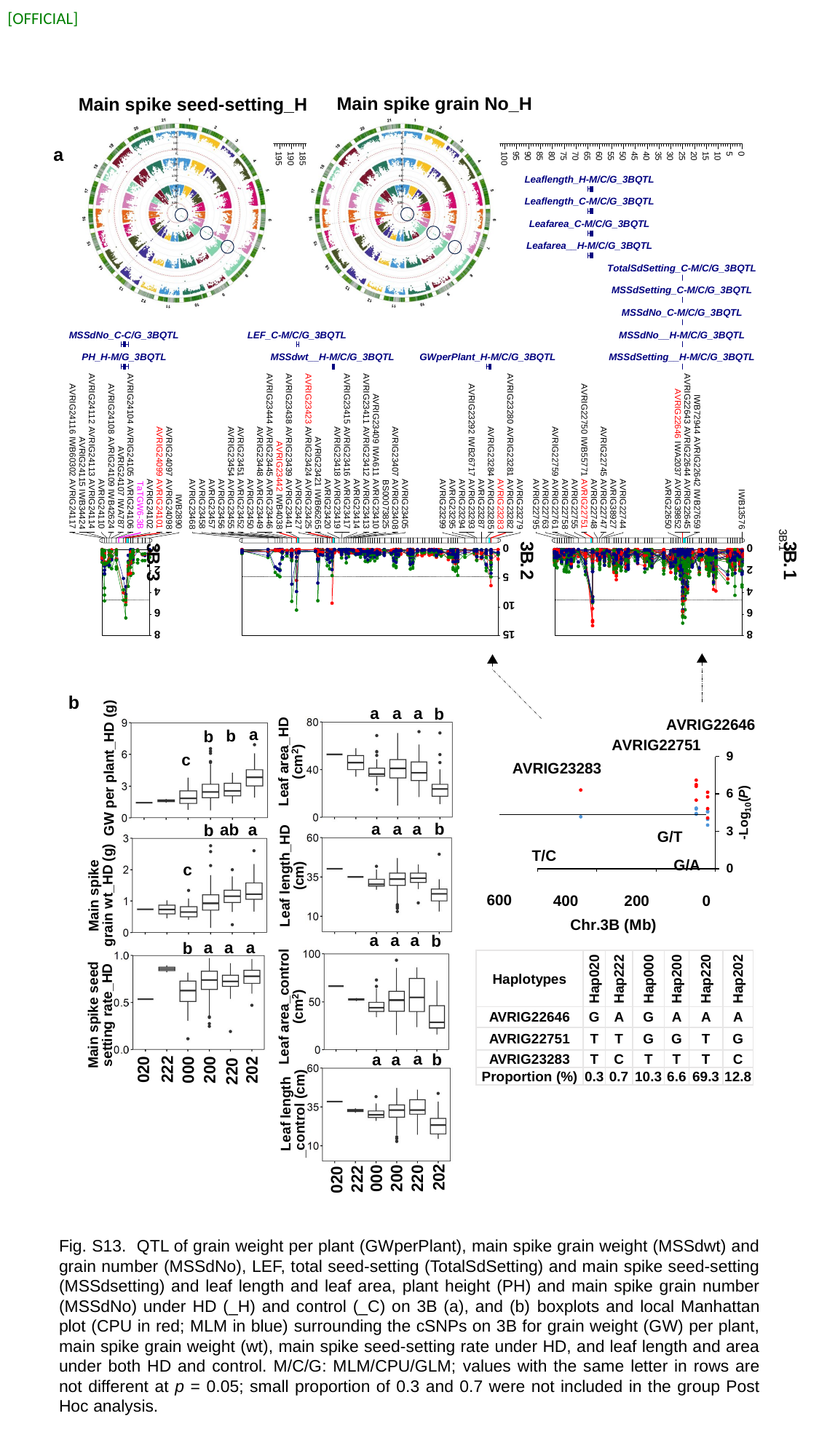

Fig. S13. QTL of grain weight per plant (GWperPlant), main spike grain weight (MSSdwt) and grain number (MSSdNo), LEF, total seed-setting (TotalSdSetting) and main spike seed-setting (MSSdsetting) and leaf length and leaf area, plant height (PH) and main spike grain number (MSSdNo) under HD (_H) and control (_C) on 3B (a), and (b) boxplots and local Manhattan plot (CPU in red; MLM in blue) surrounding the cSNPs on 3B for grain weight (GW) per plant, main spike grain weight (wt), main spike seed-setting rate under HD, and leaf length and area under both HD and control. M/C/G: MLM/CPU/GLM; values with the same letter in rows are not different at p = 0.05; small proportion of 0.3 and 0.7 were not included in the group Post Hoc analysis.

## Slide 14
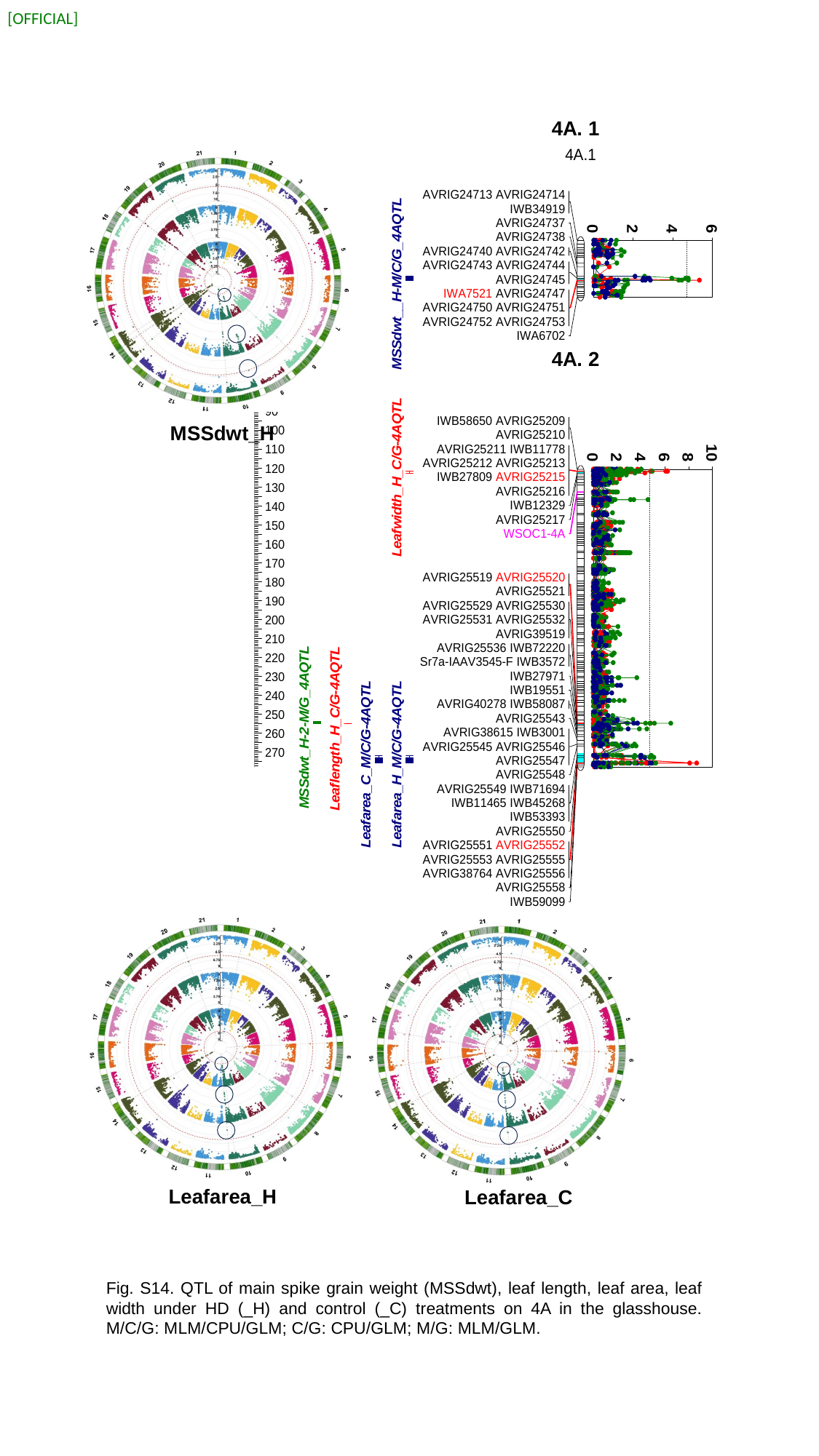

Fig. S14. QTL of main spike grain weight (MSSdwt), leaf length, leaf area, leaf width under HD (_H) and control (_C) treatments on 4A in the glasshouse. M/C/G: MLM/CPU/GLM; C/G: CPU/GLM; M/G: MLM/GLM.

## Slide 15
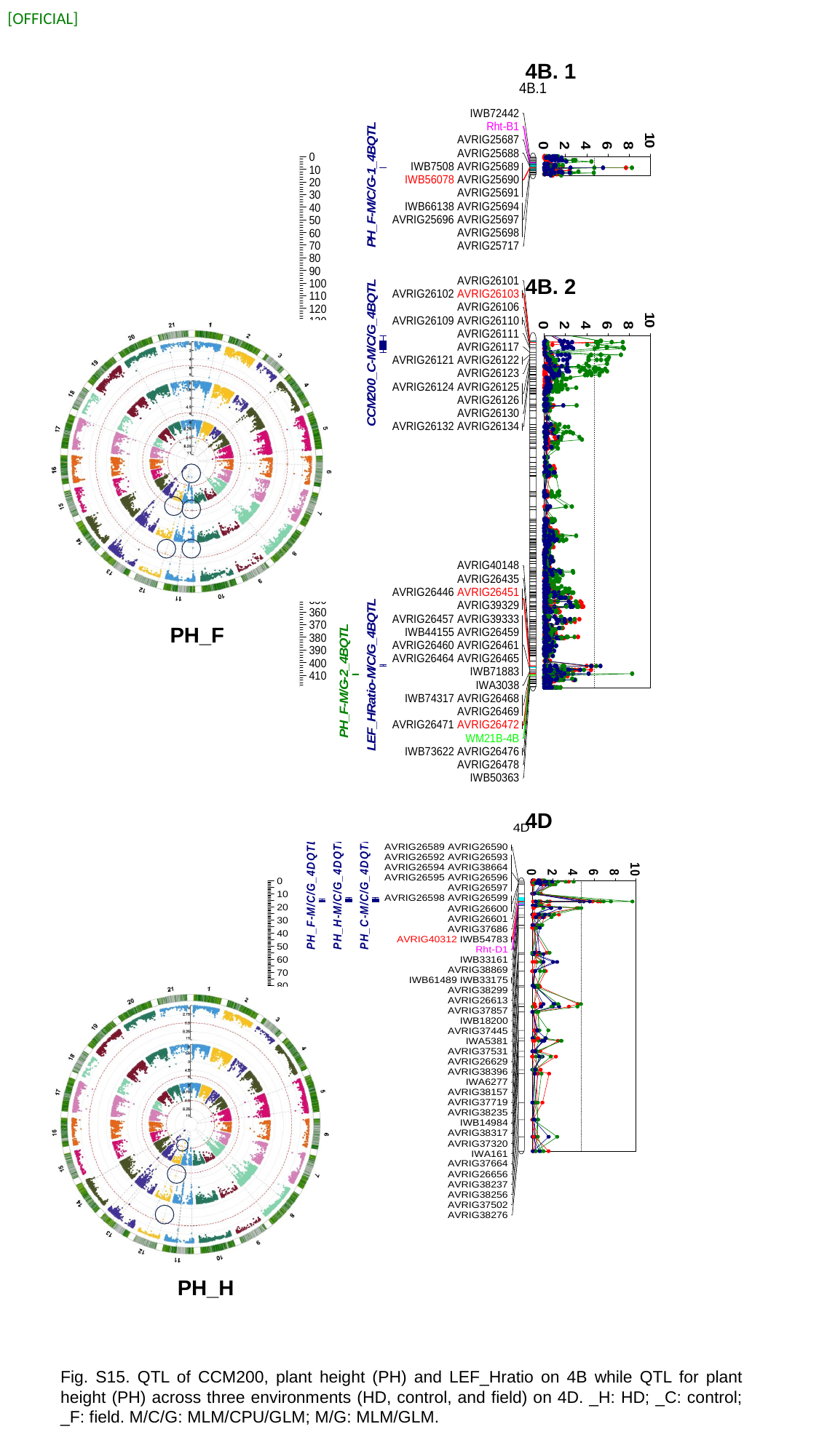

Fig. S15. QTL of CCM200, plant height (PH) and LEF_Hratio on 4B while QTL for plant height (PH) across three environments (HD, control, and field) on 4D. _H: HD; _C: control; _F: field. M/C/G: MLM/CPU/GLM; M/G: MLM/GLM.

## Slide 16
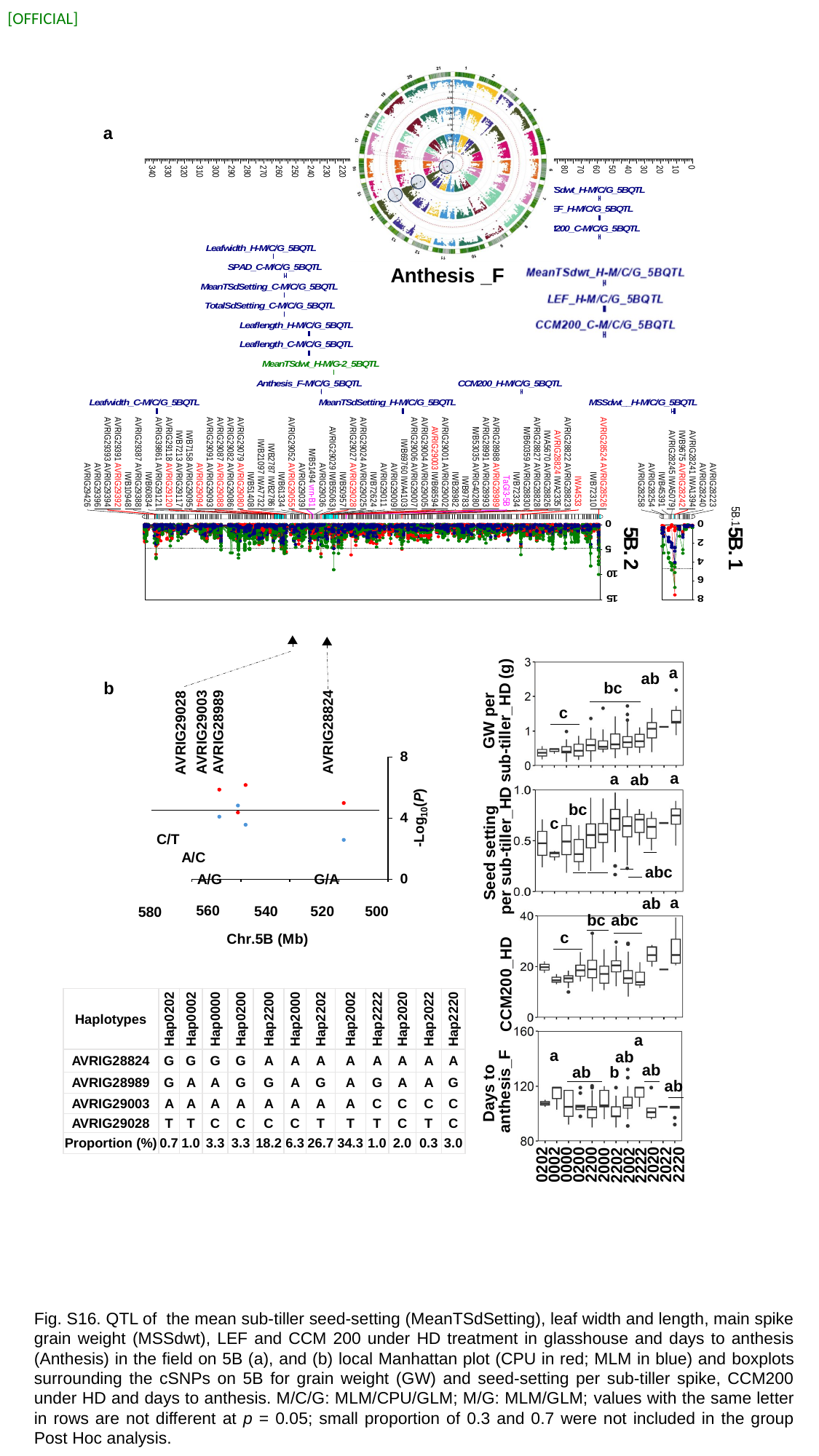

Fig. S16. QTL of the mean sub-tiller seed-setting (MeanTSdSetting), leaf width and length, main spike grain weight (MSSdwt), LEF and CCM 200 under HD treatment in glasshouse and days to anthesis (Anthesis) in the field on 5B (a), and (b) local Manhattan plot (CPU in red; MLM in blue) and boxplots surrounding the cSNPs on 5B for grain weight (GW) and seed-setting per sub-tiller spike, CCM200 under HD and days to anthesis. M/C/G: MLM/CPU/GLM; M/G: MLM/GLM; values with the same letter in rows are not different at p = 0.05; small proportion of 0.3 and 0.7 were not included in the group Post Hoc analysis.

## Slide 17
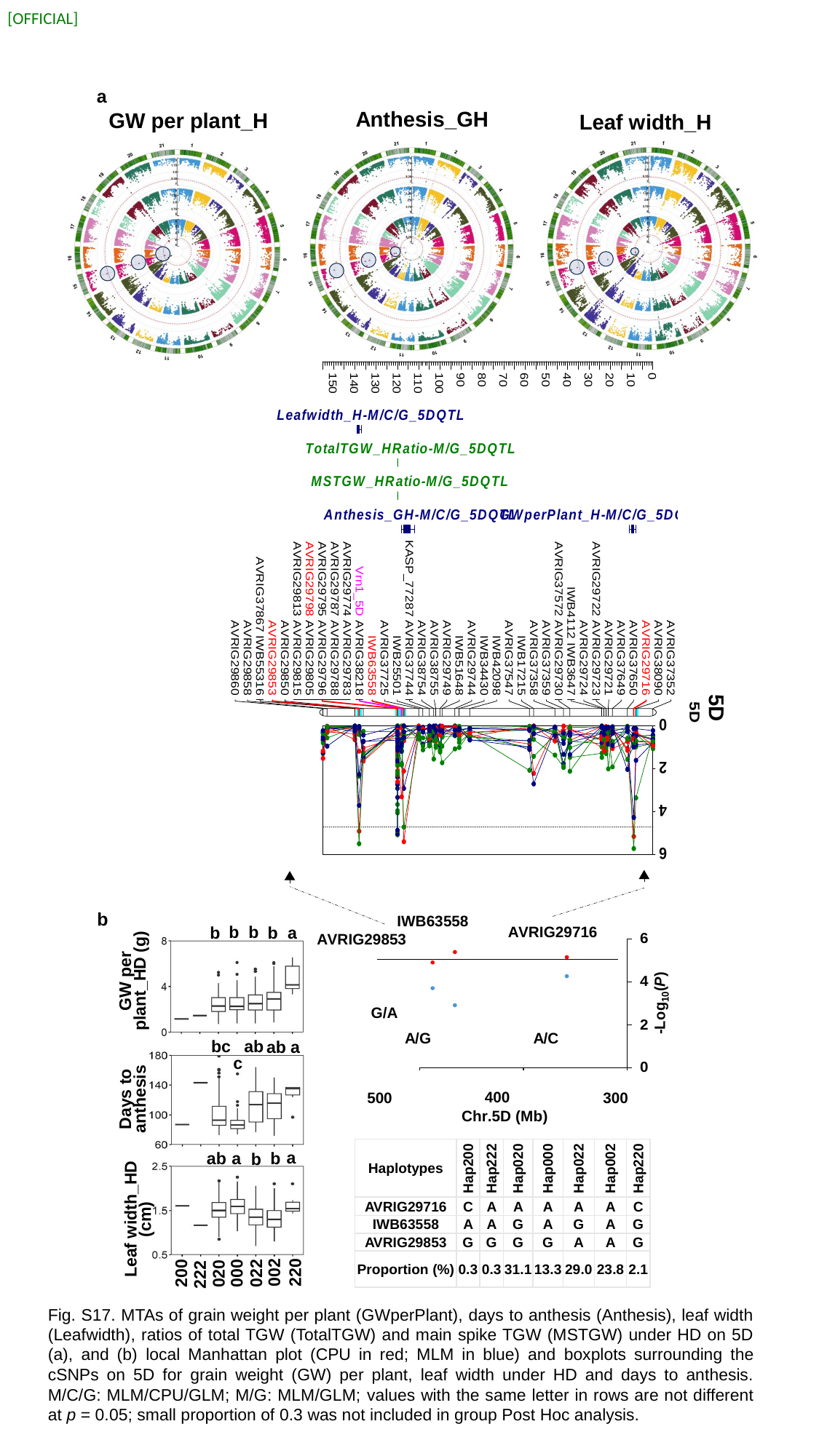

Fig. S17. MTAs of grain weight per plant (GWperPlant), days to anthesis (Anthesis), leaf width (Leafwidth), ratios of total TGW (TotalTGW) and main spike TGW (MSTGW) under HD on 5D (a), and (b) local Manhattan plot (CPU in red; MLM in blue) and boxplots surrounding the cSNPs on 5D for grain weight (GW) per plant, leaf width under HD and days to anthesis. M/C/G: MLM/CPU/GLM; M/G: MLM/GLM; values with the same letter in rows are not different at p = 0.05; small proportion of 0.3 was not included in group Post Hoc analysis.

## Slide 18
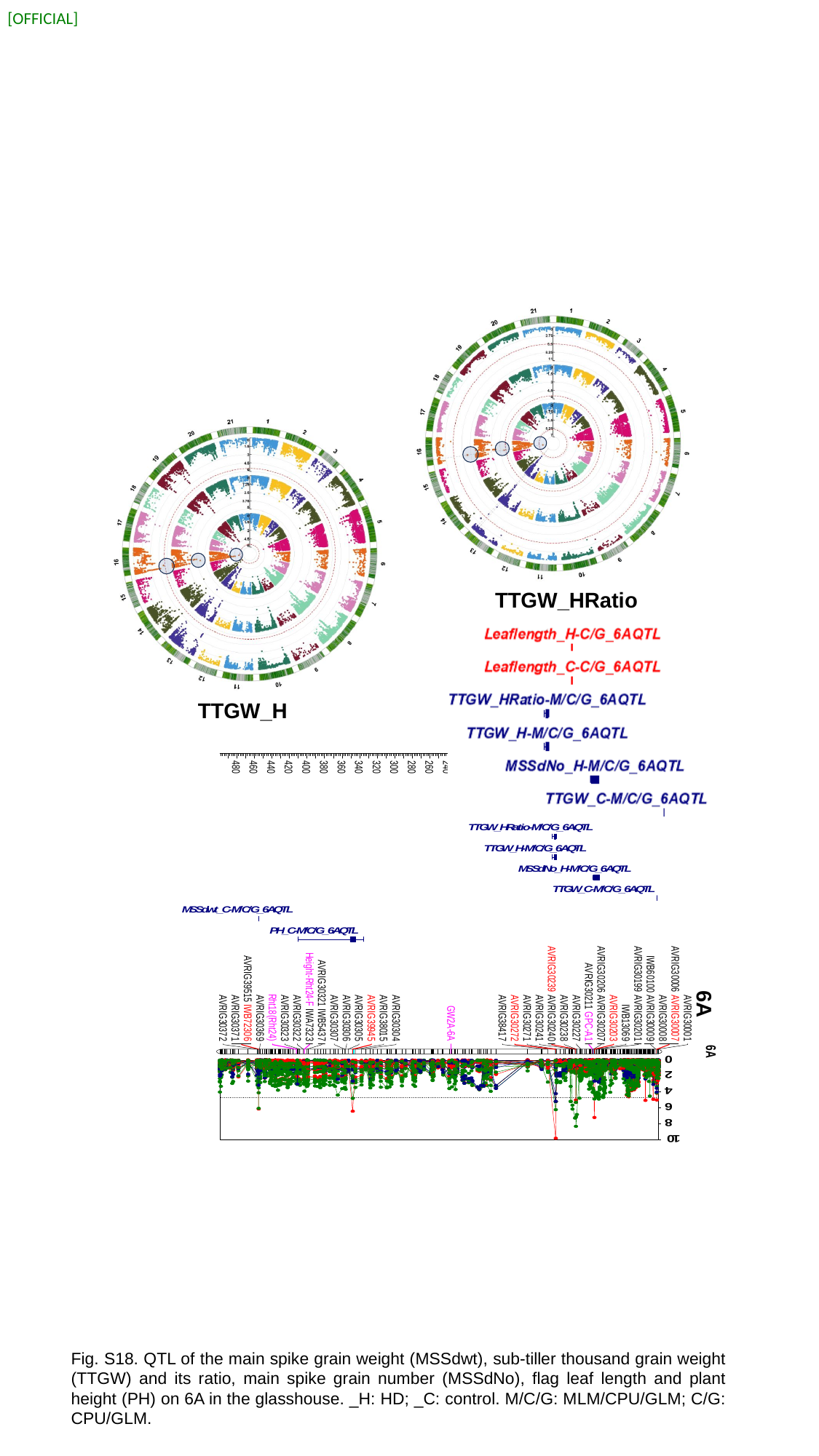

Fig. S18. QTL of the main spike grain weight (MSSdwt), sub-tiller thousand grain weight (TTGW) and its ratio, main spike grain number (MSSdNo), flag leaf length and plant height (PH) on 6A in the glasshouse. _H: HD; _C: control. M/C/G: MLM/CPU/GLM; C/G: CPU/GLM.

## Slide 19
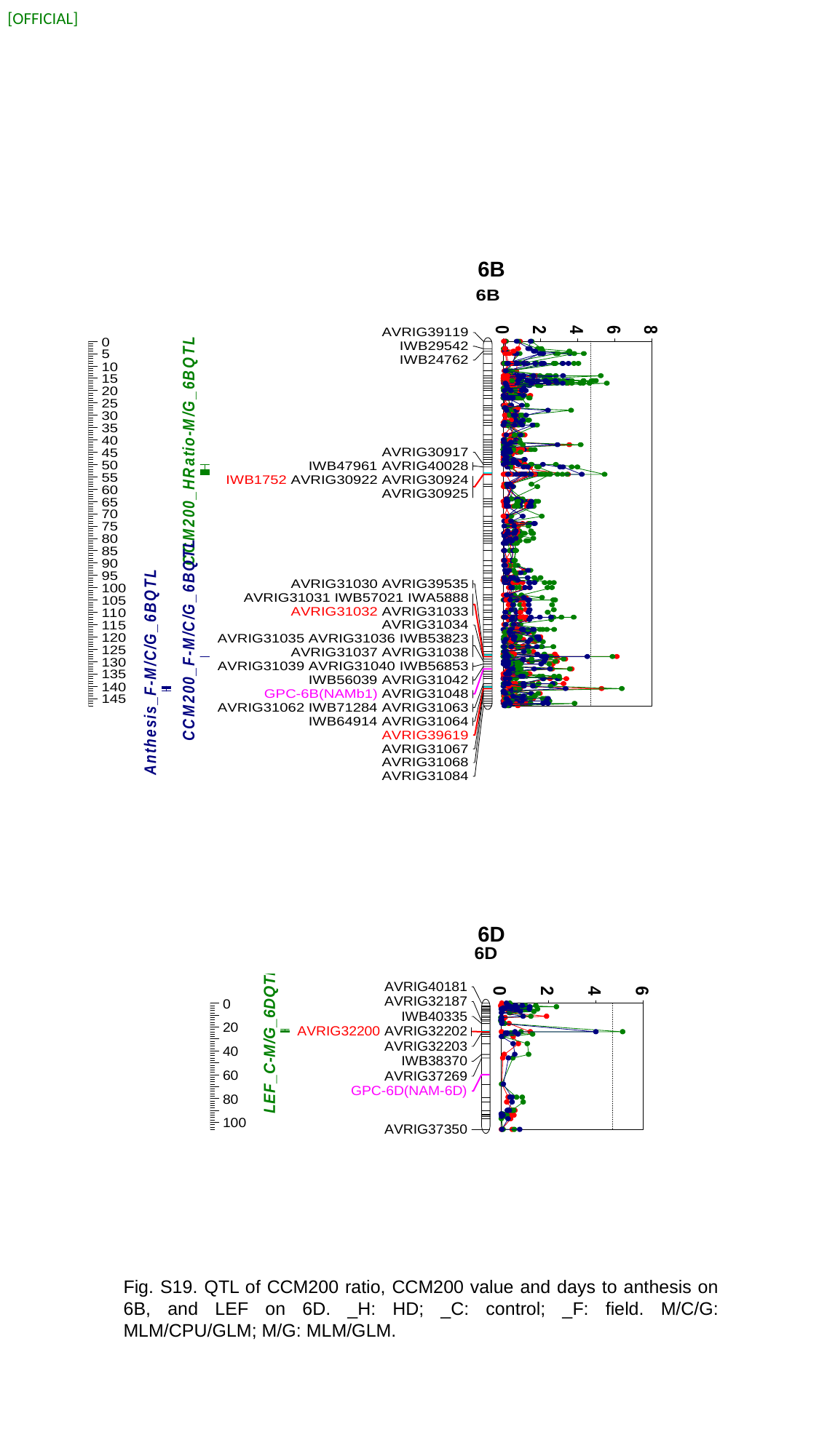

Fig. S19. QTL of CCM200 ratio, CCM200 value and days to anthesis on 6B, and LEF on 6D. _H: HD; _C: control; _F: field. M/C/G: MLM/CPU/GLM; M/G: MLM/GLM.

## Slide 20
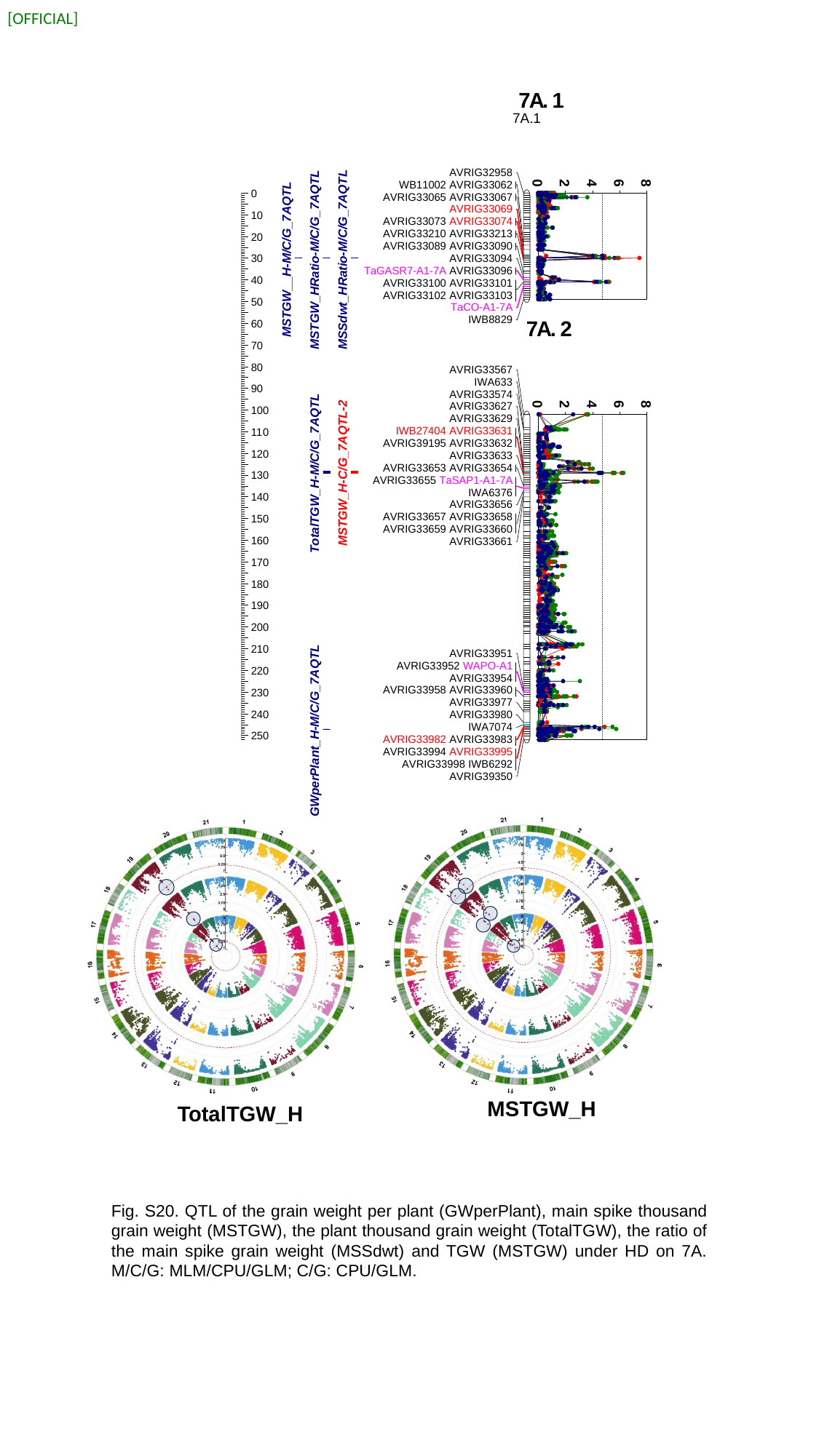

Fig. S20. QTL of the grain weight per plant (GWperPlant), main spike thousand grain weight (MSTGW), the plant thousand grain weight (TotalTGW), the ratio of the main spike grain weight (MSSdwt) and TGW (MSTGW) under HD on 7A. M/C/G: MLM/CPU/GLM; C/G: CPU/GLM.

## Slide 21
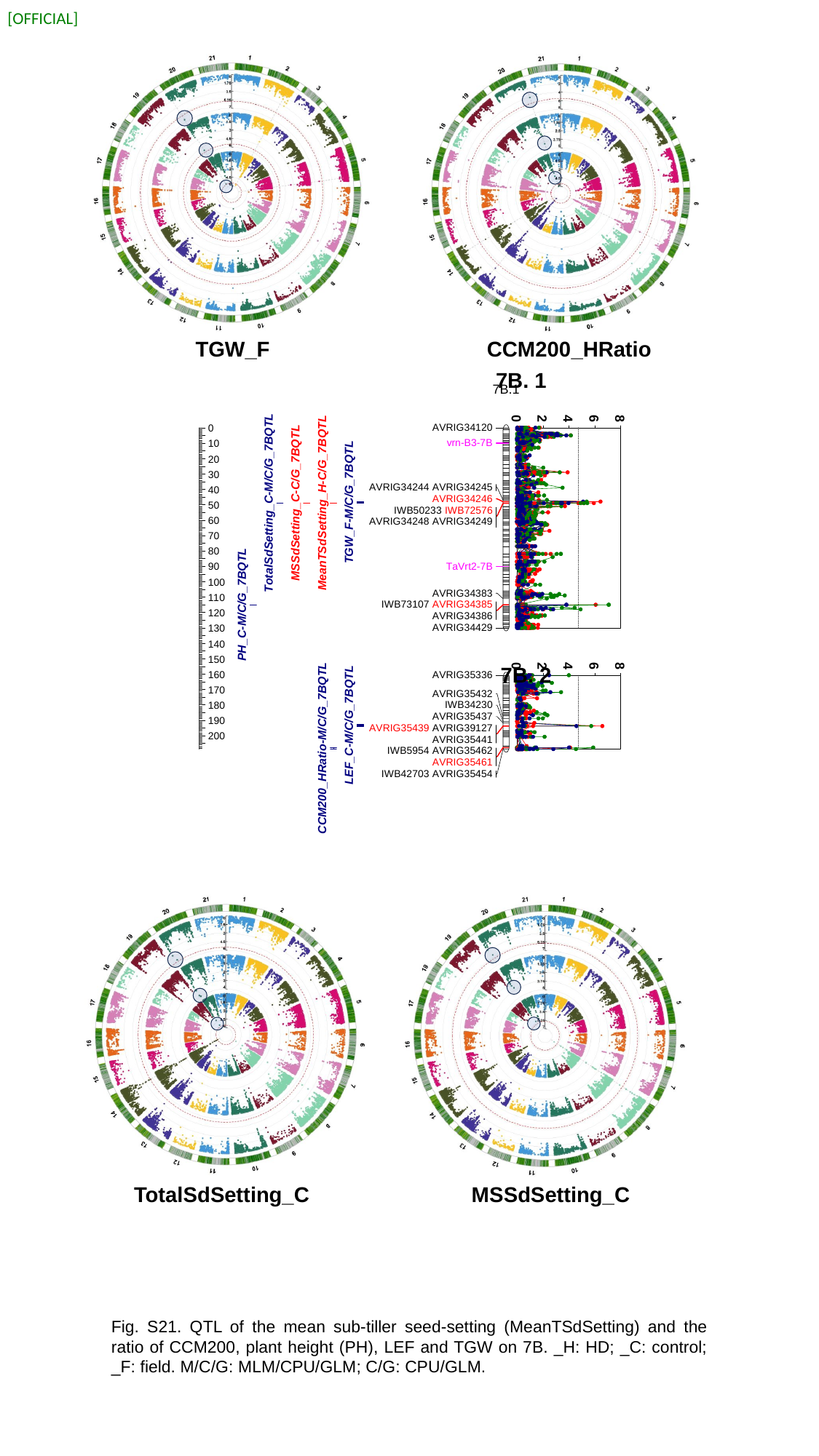

Fig. S21. QTL of the mean sub-tiller seed-setting (MeanTSdSetting) and the ratio of CCM200, plant height (PH), LEF and TGW on 7B. _H: HD; _C: control; _F: field. M/C/G: MLM/CPU/GLM; C/G: CPU/GLM.

## Slide 22
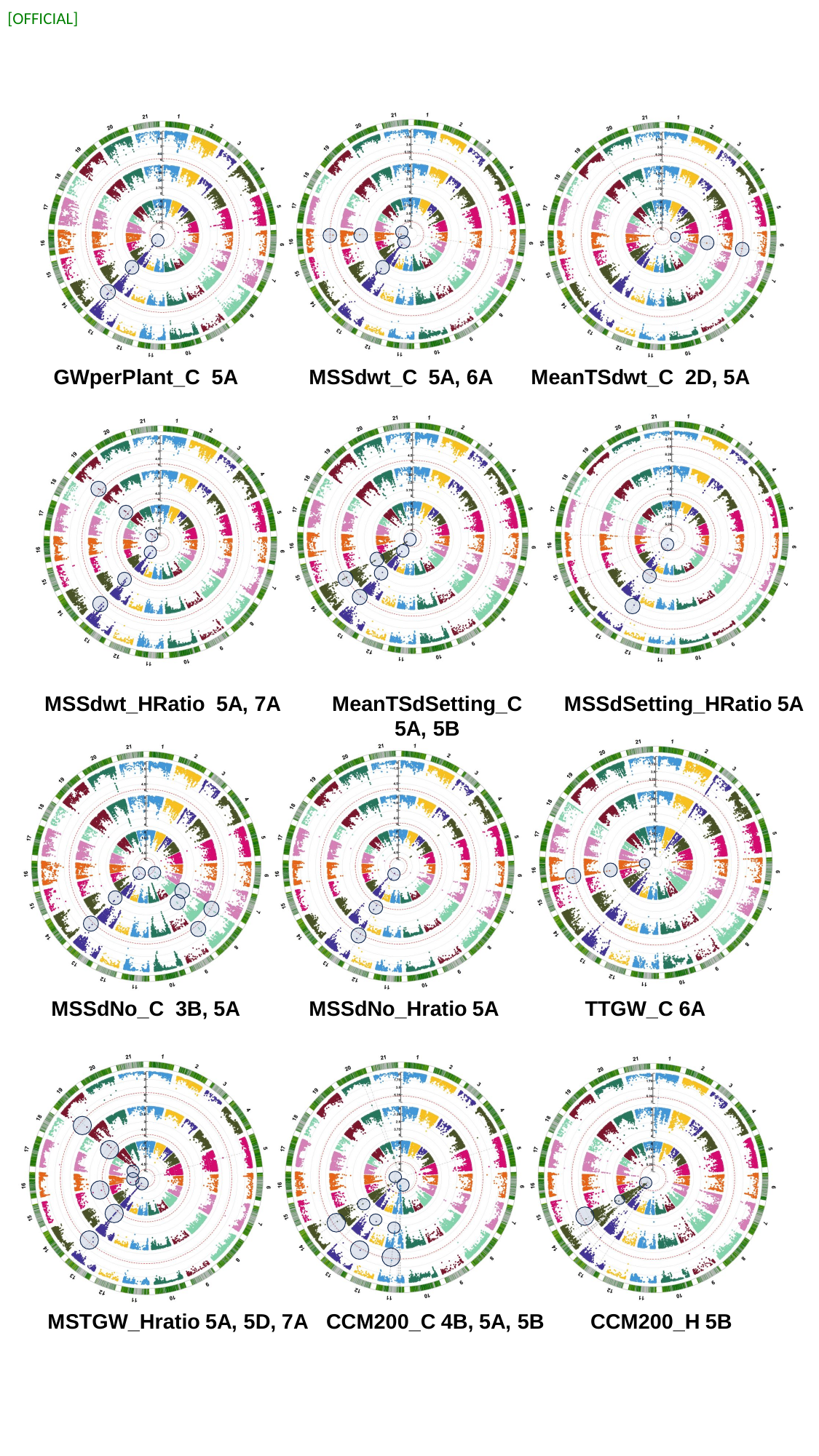

## Slide 23
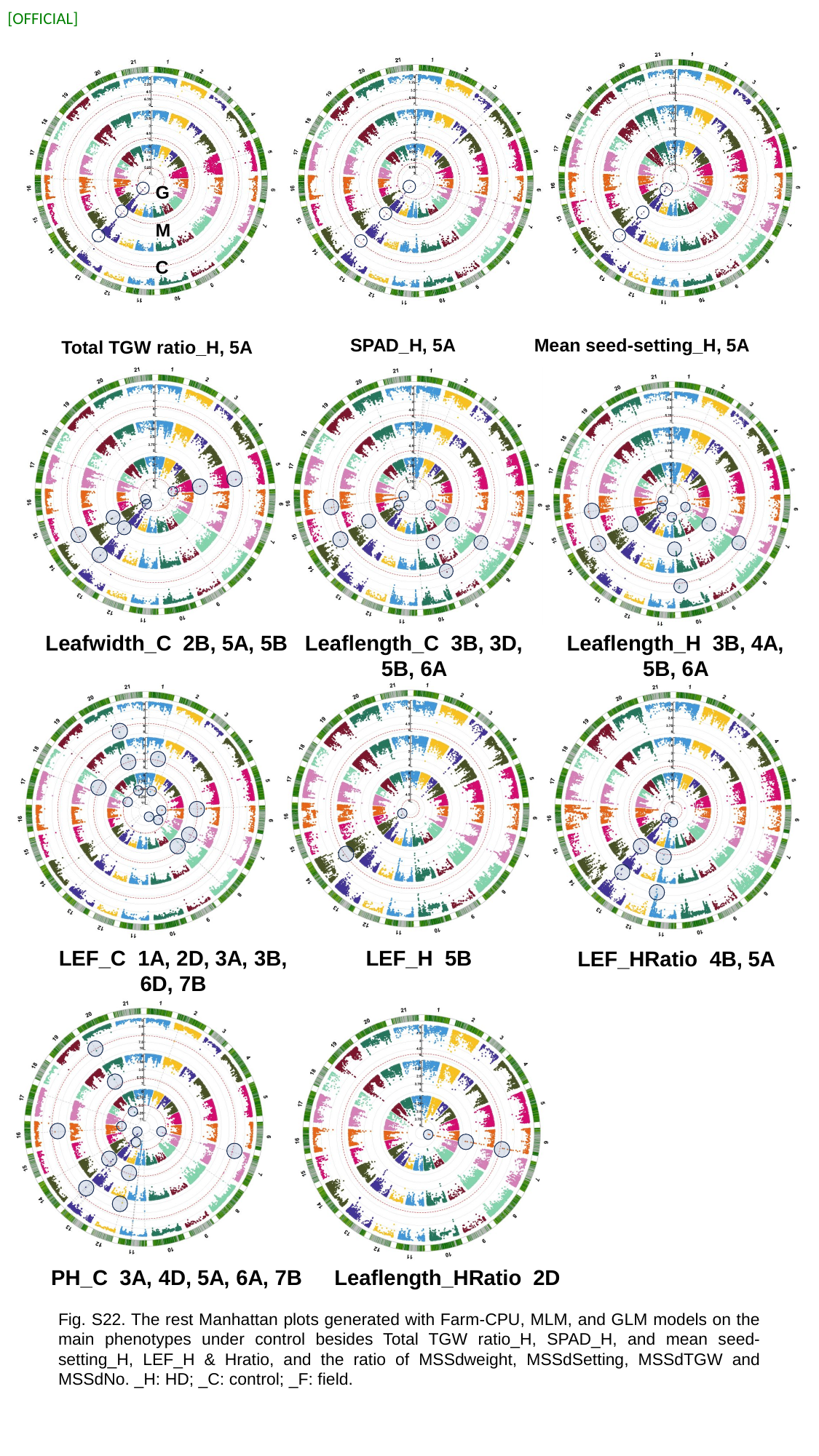

Fig. S22. The rest Manhattan plots generated with Farm-CPU, MLM, and GLM models on the main phenotypes under control besides Total TGW ratio_H, SPAD_H, and mean seed-setting_H, LEF_H & Hratio, and the ratio of MSSdweight, MSSdSetting, MSSdTGW and MSSdNo. _H: HD; _C: control; _F: field.
